# Supplementary material for: Synthesis and Glycosidase Inhibition of Broussonetine M and Its Analogues
Source: Molecules. 2019 Oct 15;24(20):3712. doi: 10.3390/molecules24203712 (PMC6832352; doi:10.3390/molecules24203712)
Supplement: Supplementary file 1 [file molecules-24-03712-s001.pdf]

Synthesis and Glycosidase Inhibition of Broussonetine M and Its Analogues

Qing-Kun Wu [1,4], Kyoko Kinami [2], Atsushi Kato [2,*], Yi-Xian Li [1,4], Yue-Mei Jia [1,4], George W. J. Fleet [3,5], and Chu-Yi Yu [1,4,5,*]

[1] Beijing National Laboratory for Molecular Science (BNLMS), CAS Key Laboratory of Molecular Recognition and Function, Institute of Chemistry, Chinese Academy of Sciences, Beijing 100190, China; lsqkwu@iccas.ac.cn (Q.-K.W.); tamarali@iccas.ac.cn (Y.-X.L.); jiayuemei@iccas.ac.cn (Y.-M.J.); yucy@iccas.ac.cn (C.-Y.Y.)

[2] Department of Hospital Pharmacy, University of Toyama, 2630 Sugitani, Toyama 930-0194, Japan; kimagure1242@yahoo.co.jp (K.K.); kato@med.u-toyama.ac.jp (A.K.)

[3] Chemistry Research Laboratory, Department of Chemistry, University of Oxford, Mansfield Road, Oxford, post code OX13TA, UK; george.fleet@chem.ox.ac.uk (G.W.J.F.)

[4] University of Chinese Academy of Sciences, Beijing 100049, China

[5] National Engineering Research Center for Carbohydrate Synthesis, Jiangxi Normal University, Nanchang 330022, PR China

* Correspondence: kato@med.u-toyama.ac.jp (A.K.); yucy@iccas.ac.cn (C.-Y.Y.); Tel.: +86-010-626-12893 (C.-Y.Y.)

Supporting Information

Contents

1. Comparison of NMR Data of the synthetic broussonetine M (**3**) with natural product and the synthetic broussonetine M in literature........S2
2. Copies of NMR spectra for all new compounds........S6
3. HPLC Analysis of Compounds **15** and ***ent*-15**........S6

1. Comparison of NMR data of the synthetic broussonetine M (3) with natural product and the synthetic broussonetine M in literature

Table S1. Comparison of ^{1}H NMR of the synthetic broussonetine M (**3**) with natural product and the synthetic broussonetine M of J. Alberto Marco

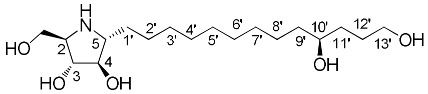

		**Natural Product[b]**	**Synthetic Broussonetine M (lit.)[c]**	**Compound 3**
		(500 MHz, C_5D_5N)	(500 MHz, C_5D_5N)	(500 MHz, C_5D_5N)
**Pyrrolidine Ring**	CH_2OH	4.15 (dd, *J* = 11.0, 5.9 Hz), 4.22 (dd, *J* = 11.0, 5.9 Hz)	4.45 (dd, *J* = 22.5, 5.5 Hz), 4.41 (dd, *J* = 11.5, 5 Hz)	4.49–4.42 (m, 2H)
	2	3.75 (m)	4.26 (m)	4.31–4.28 (m, 1H)
	3	4.62 (t, *J* = 6.4 Hz)	4.90 (t, *J* = 7 Hz)	4.92 (t, *J* = 6.7 Hz)
	4	4.36 (t, *J* = 6.4 Hz)	4.65 (t, *J* = 7 Hz)	4.67 (t, *J* = 7.1 Hz)
	5	3.45 (m)	4.05 (br, q, *J* = 7.5 Hz)	4.09–4.05 (m, 1H)
**Side Chain**	1’	1.70 (m)[a], 2.00 (m)[a]	2.15–2.10 (m, 1H), 2.05–2.00 (m, 1H)	2.15–2.09 (m, 1H), 2.03–1.97 (m, 1H)
	2’	1.66–1.16	1.90–1.50 (m)	1.90–1.79 (m)[a], 1.74–1.56 (m)[a]
	3’	1.66–1.16	1.40–1.15 (m)[a]	1.33–1.07 (m)
	4’	1.66–1.16	1.40–1.15 (m)[a]	1.33–1.07 (m)
	5’	1.66–1.16	1.40–1.15 (m)[a]	1.33–1.07 (m)
	6’	1.66–1.16	1.40–1.15 (m)[a]	1.33–1.07 (m)
	7’	1.66–1.16	1.40–1.15 (m)[a]	1.33–1.07 (m)
	8’	1.66–1.16	1.90–1.50 (m)[a]	1.74–1.56 (m)[a], 1.48–1.45 (m, 1H)
	9’	1.63 (m)[a], 1.70 (m)[a]	1.90–1.50 (m)[a]	1.74–1.56 (m)[a]
	10’	3.90 (m)	3.90 (m)	3.92–3.87 (m 1H)
	11’	1.86 (m)	1.90–1.50 (m)[a]	1.90–1.79 (m)[a]
	12’	2.00 (m)[a], 2.12 (m)	2.30–2.25(br, m)	2.34–2.29 (m, 2H)
	13’	3.95 (t, *J* = 6.4 Hz)	3.96 (t, *J* = 6.5 Hz)	3.96 (t, *J* = 6.4 Hz)

a) Overlapped signals; b) Shibano, M.; Tsukamoto, D.; Fujimoto, R.; Masui, Y.; Sugimoto, H.; Kusano, G. Studies on the Constituents of Broussonetia Species. VII. Four New Pyrrolidine Alkaloids, Broussonetines M, O, P, and Q, as Inhibitors of Glycosidase, from Broussonetia kazinoki SIEB. *Chem. Pharm. Bull.* **2000**, *48*, 1281-1285; c) Ribes, C.; Falomir, E.; Murga, J.; Carda, M.; Alberto Marco, J. Convergent, stereoselective syntheses of the glycosidase inhibitors broussonetines D and M. *Org. Biomol. Chem.* **2009**, *7*, 1355-1360.

Table S2. Comparison of ^{13}C NMR of the synthetic broussonetine M (**3**) with natural product and the synthetic broussonetine M of J. Alberto Marco

		Natural Product[b]	Synthetic Broussonetine M (lit.)[c]	Compound 3
		(500 MHz, C_5D_5N)	(500 MHz, C_5D_5N)	(500 MHz, C_5D_5N)
**Pyrrolidine Ring**	CH_2OH	63.45	59.2	59.7
	2	64.17	65.0	65.2
	3	80.23	76.2	76.7
	4	84.01	80.3	80.7
	5	62.01	62.7	63.0
**Side Chain**	1'	35.44	30.2	30.3
	2'–8'	30.10	29.9	29.9
		30.07	29.7	29.8
		29.94	29.5	29.6
		29.88	29.4	29.5
		29.83	29.3	29.4
		27.22	26.6	26.7
		26.29	26.1	26.3
	9'	38.27	38.3	38.5
	10'	70.94	70.8	70.9
	11'	34.93	35.1	35.2
	12'	30.17	31.5	31.9
	13'	62.34	62.3	62.4

a) Shibano, M.; Tsukamoto, D.; Fujimoto, R.; Masui, Y.; Sugimoto, H.; Kusano, G. Studies on the Constituents of Broussonetia Species. VII. Four New Pyrrolidine Alkaloids, Broussonetines M, O, P, and Q, as Inhibitors of Glycosidase, from Broussonetia kazinoki SIEB. *Chem. Pharm. Bull.* **2000**, *48*, 1281-1285; b) Ribes, C.; Falomir, E.; Murga, J.; Carda, M.; Alberto Marco, J. Convergent, stereoselective syntheses of the glycosidase inhibitors broussonetines D and M. *Org. Biomol. Chem.* **2009**, *7*, 1355-1360.

Copies of NMR spectra for all new compounds

Compound 21

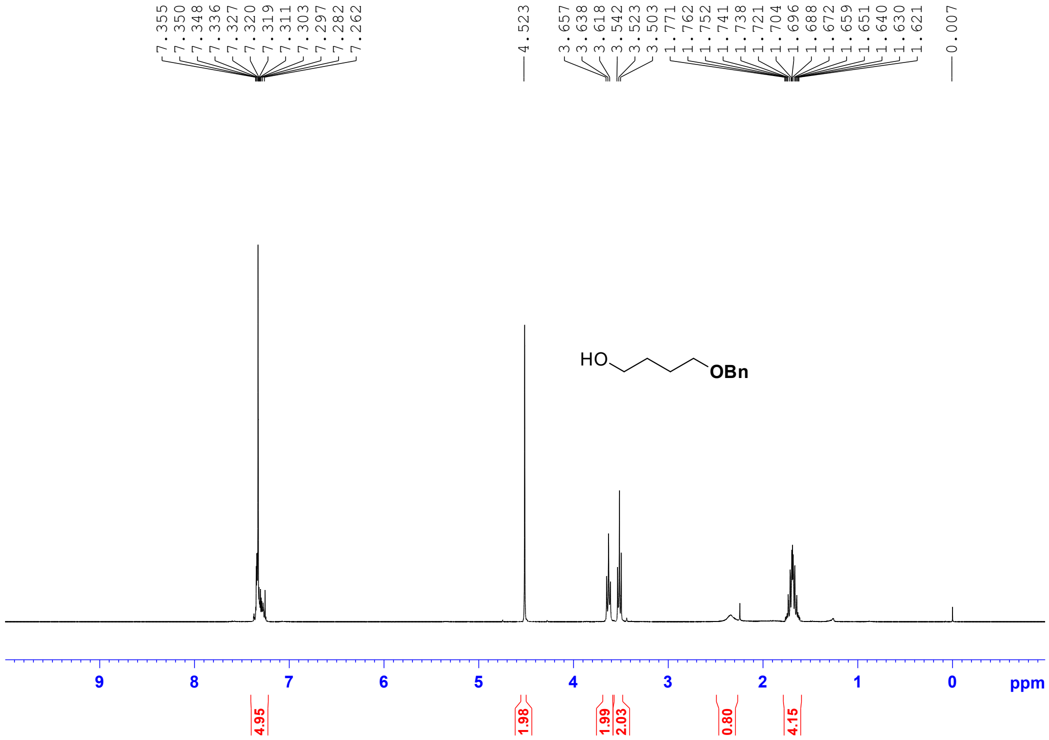

Figure S1

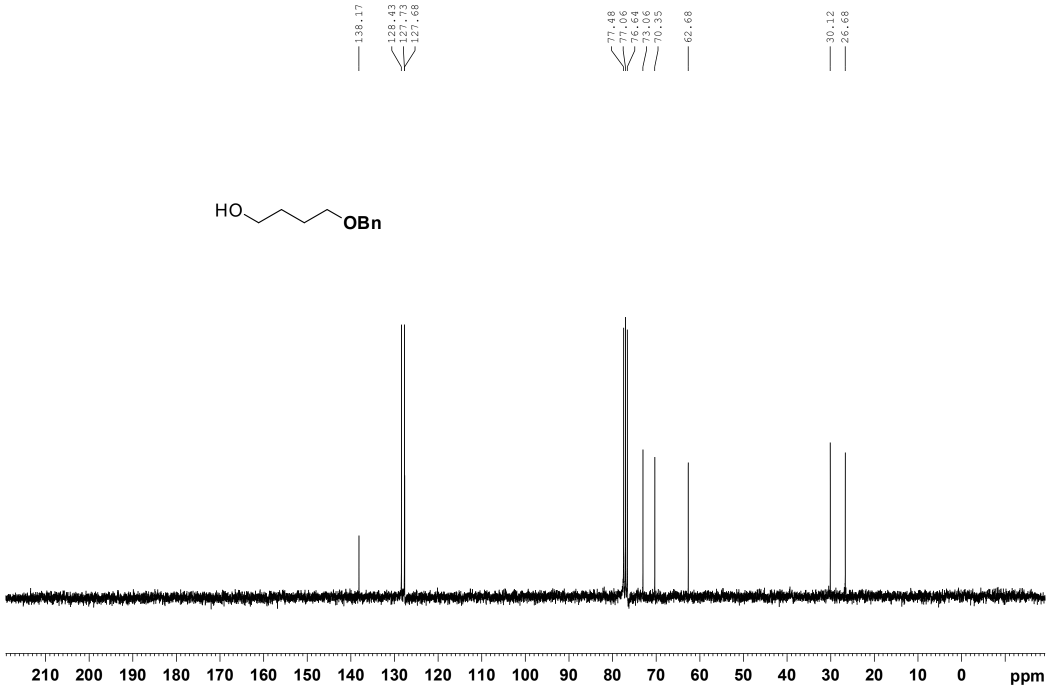

Figure S2

Compound 16

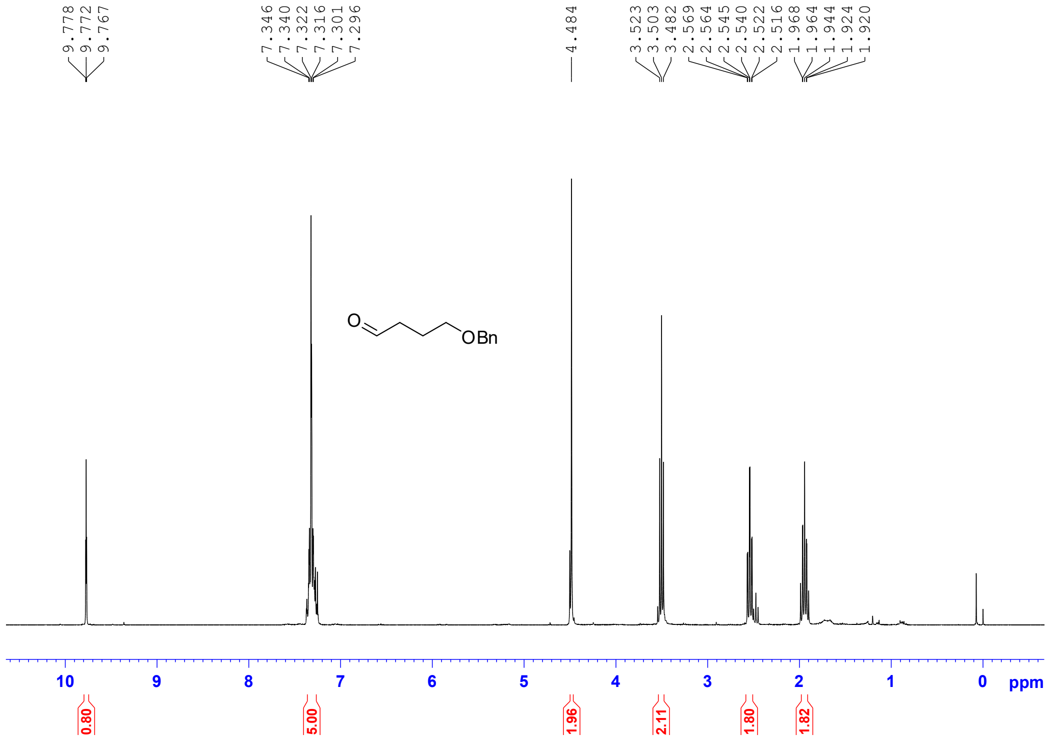

Figure S3

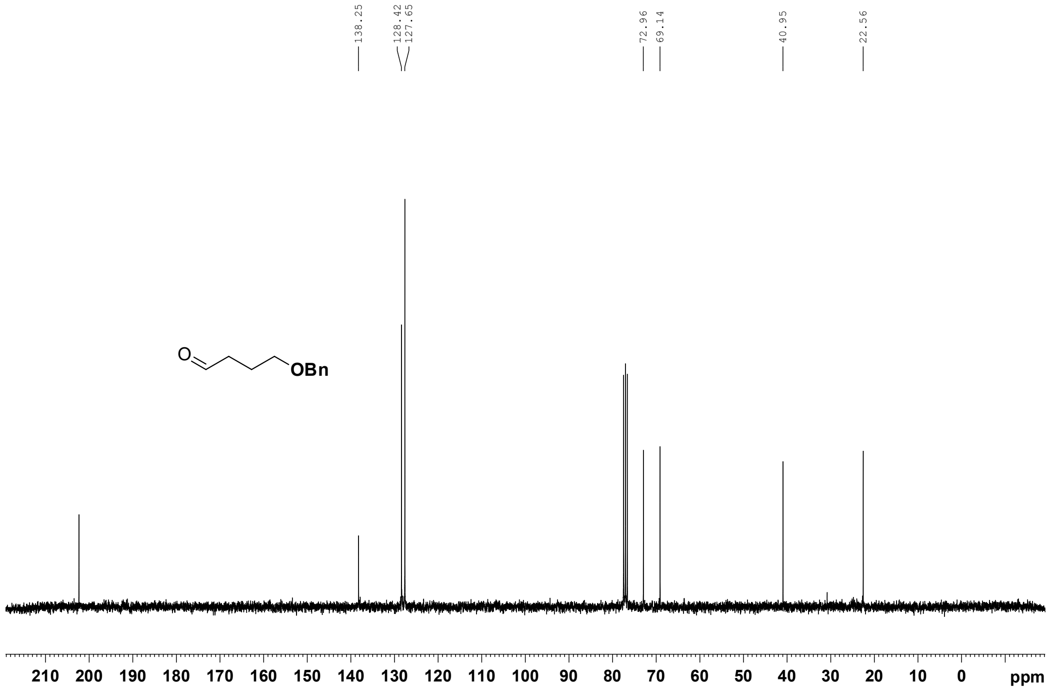

Figure S4

Compound *ent*-15

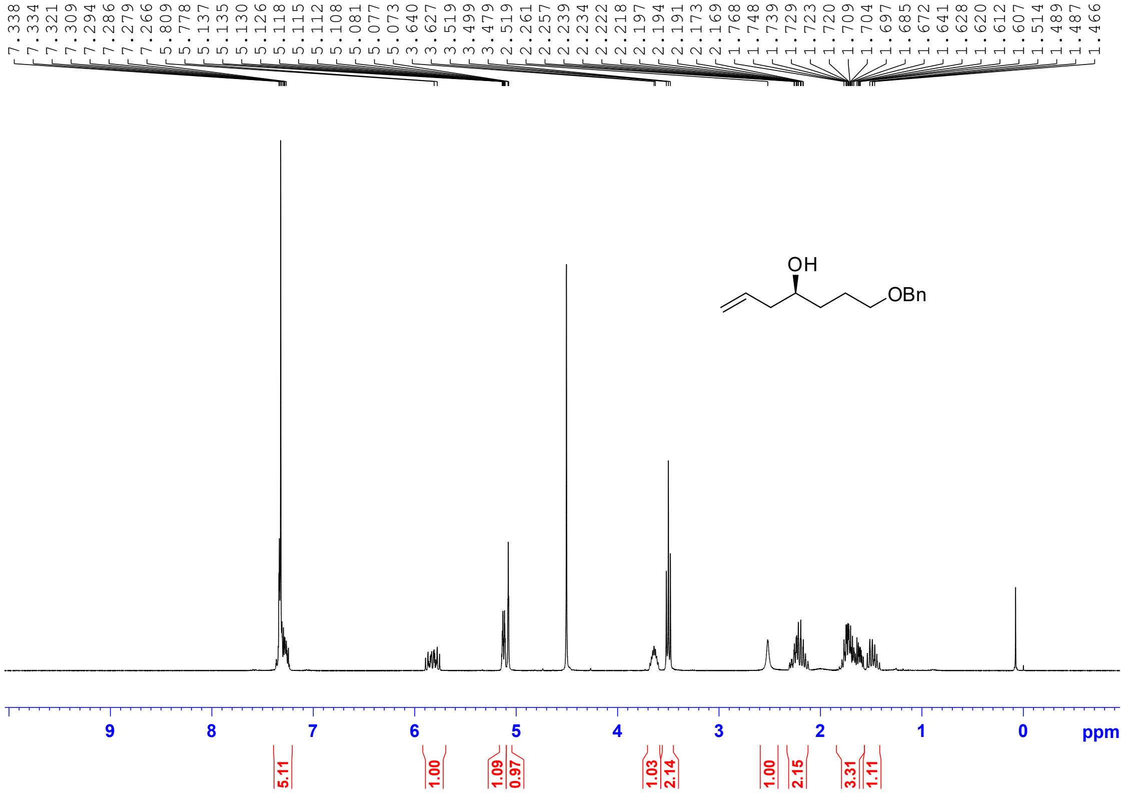

Figure S5

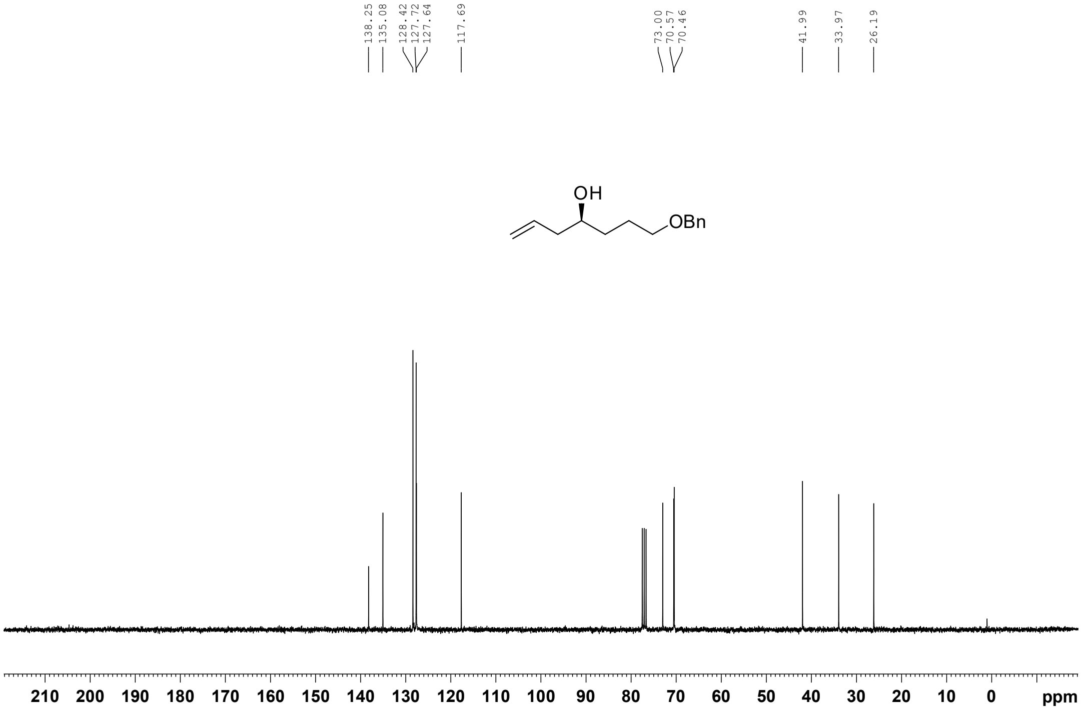

Figure S6

Compound 15

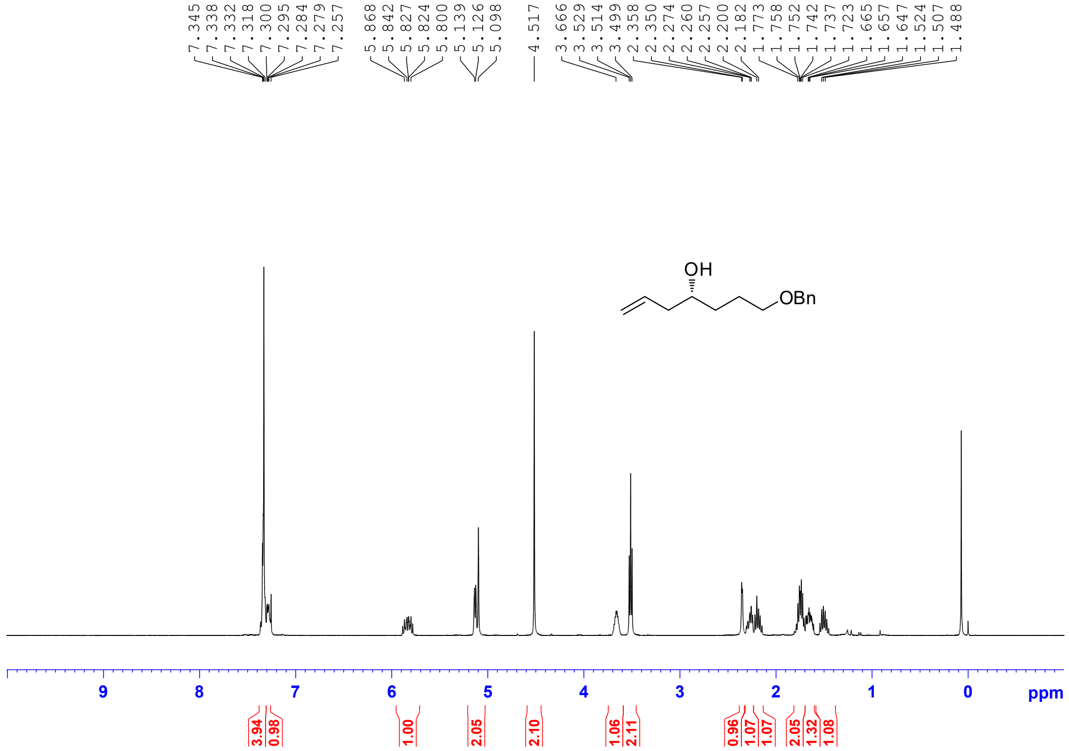

Figure S7

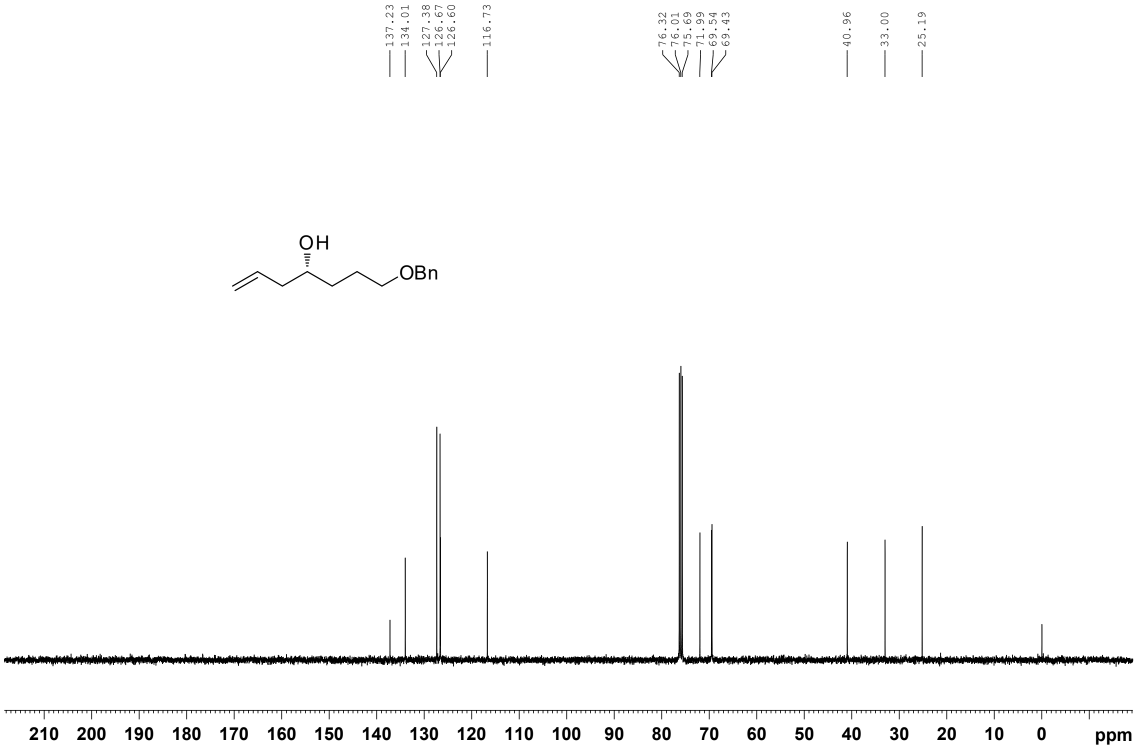

Figure S8

Compound 19

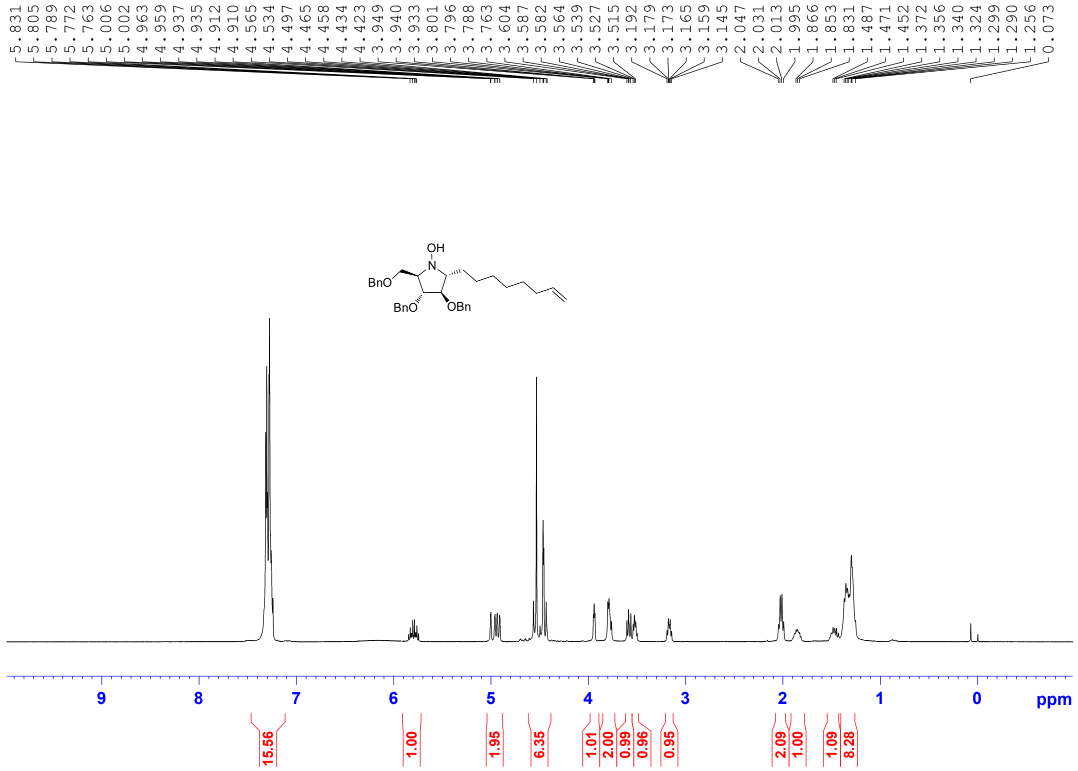

Figure S9

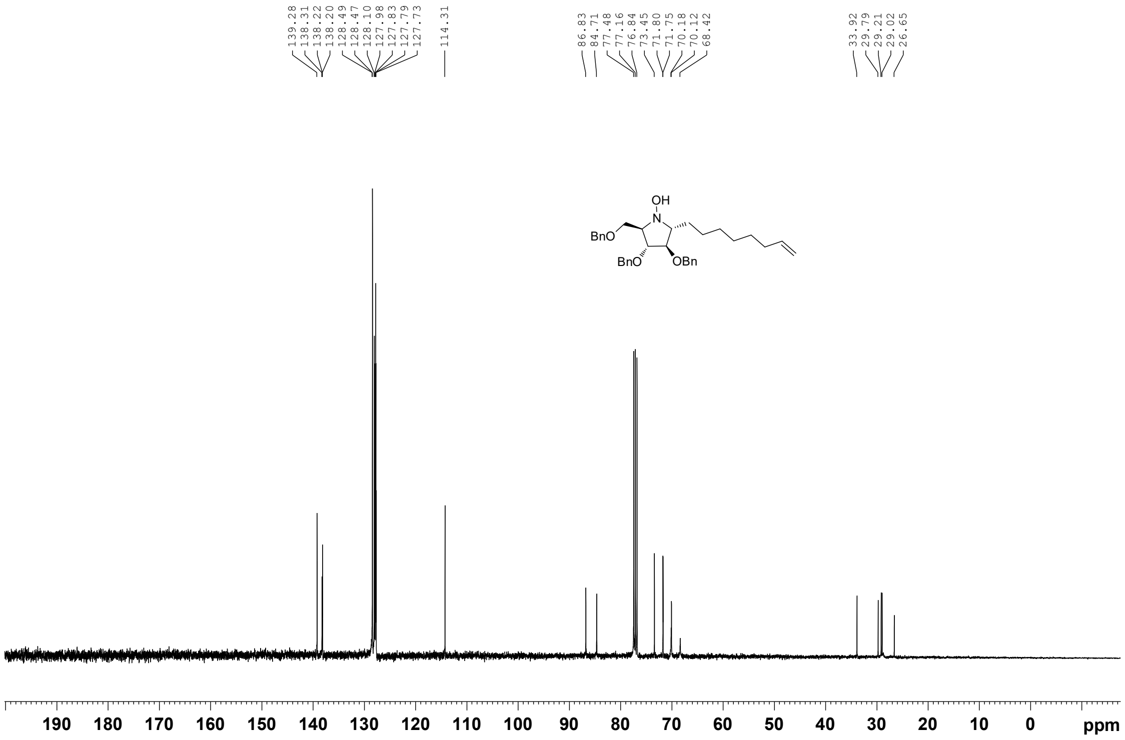

Figure S10

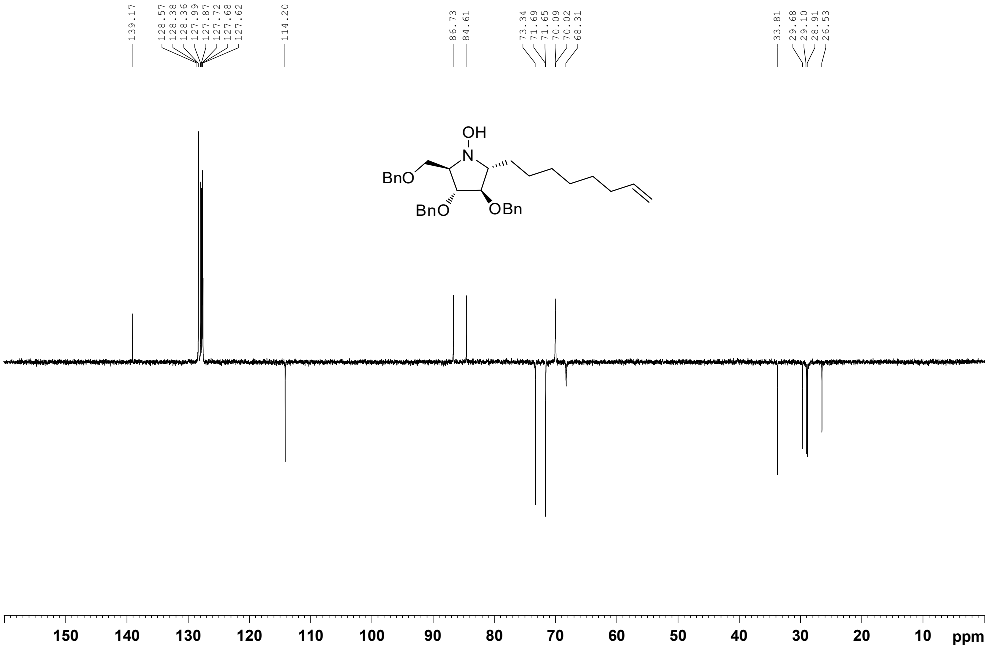

Figure S11

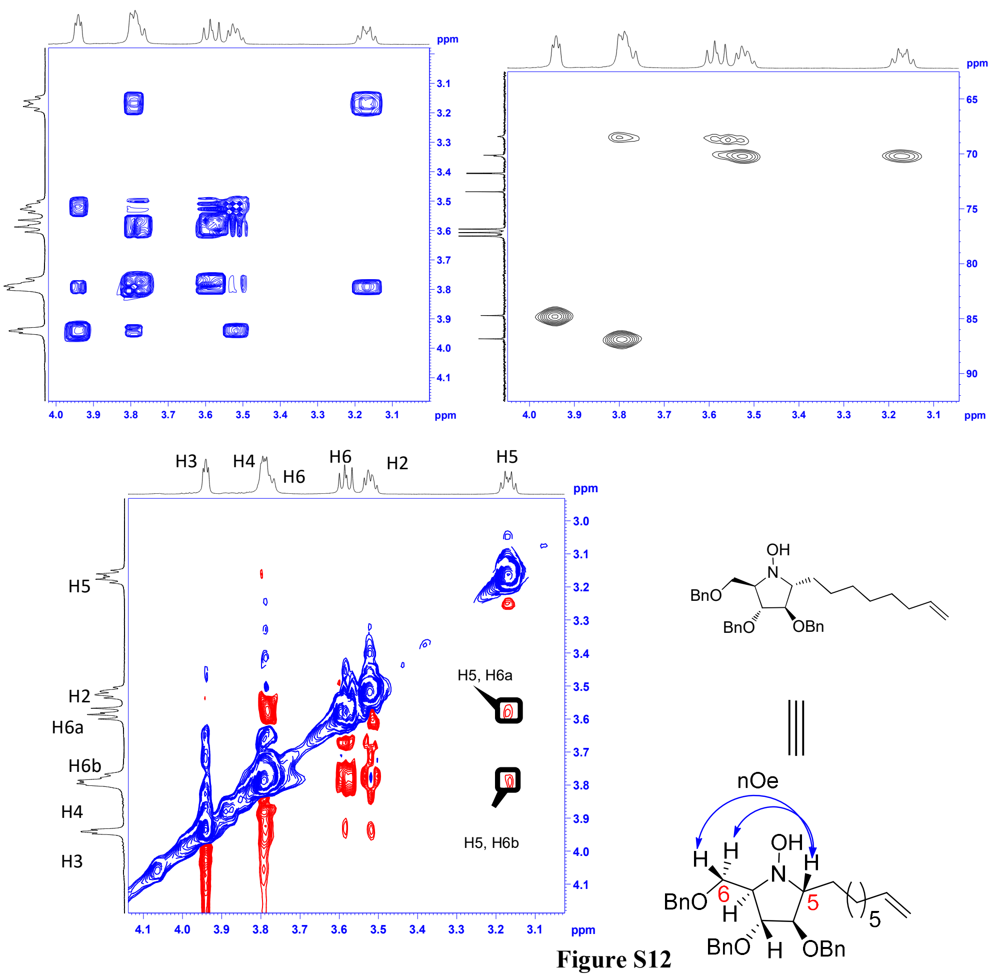

Figure S12

Compound *ent*-19

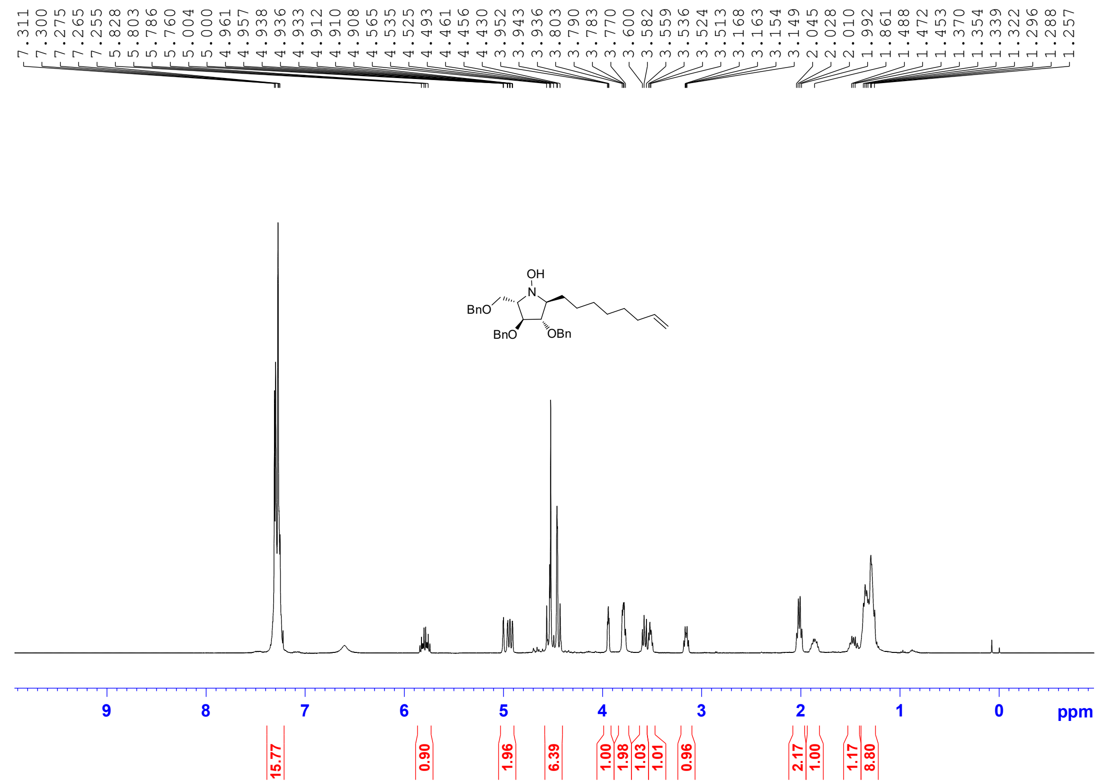

Figure S13

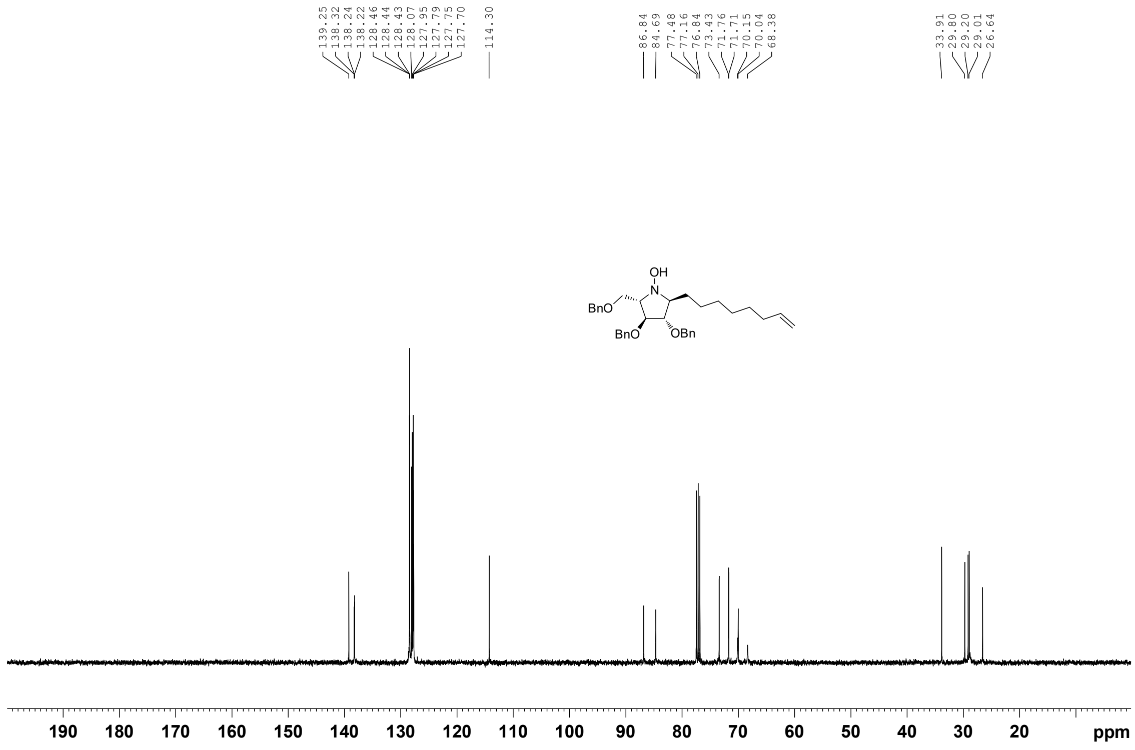

Figure S14

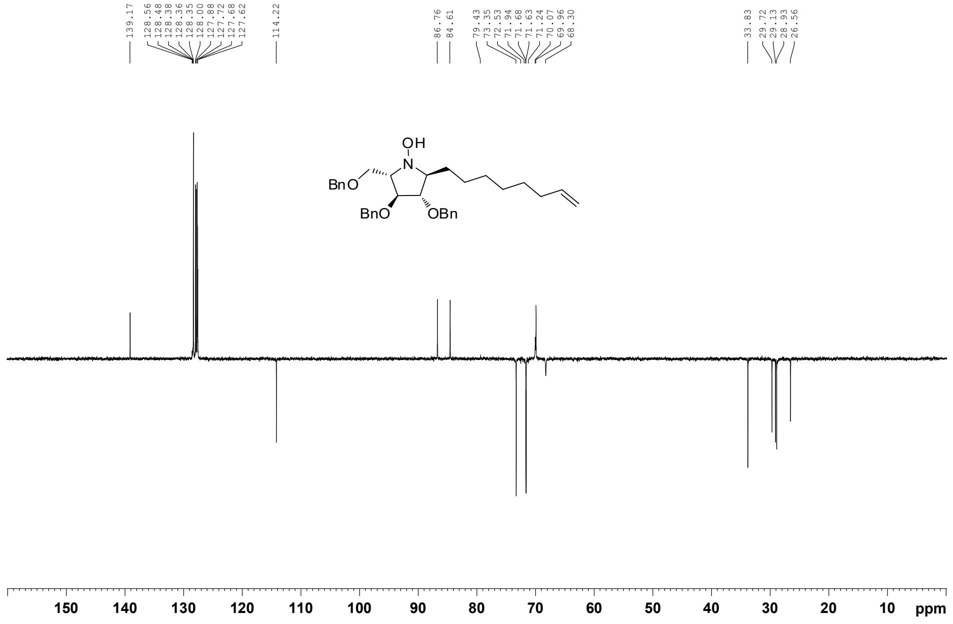

Figure S15

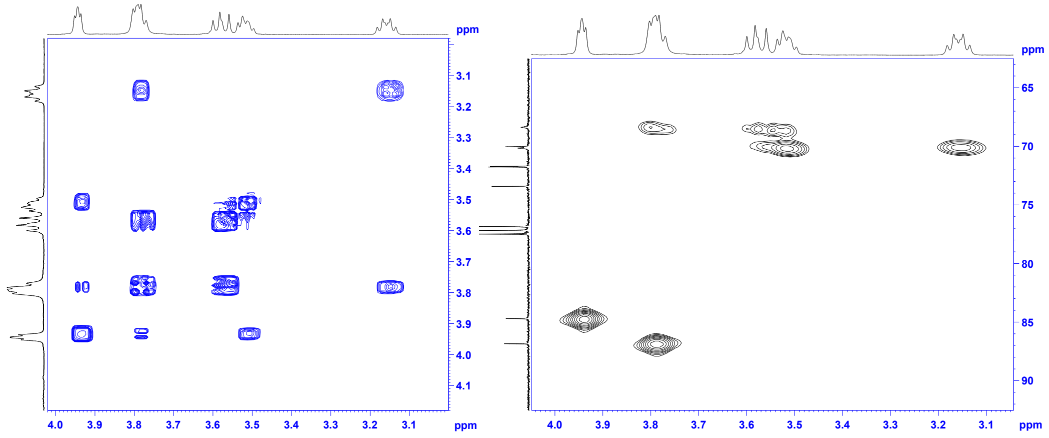

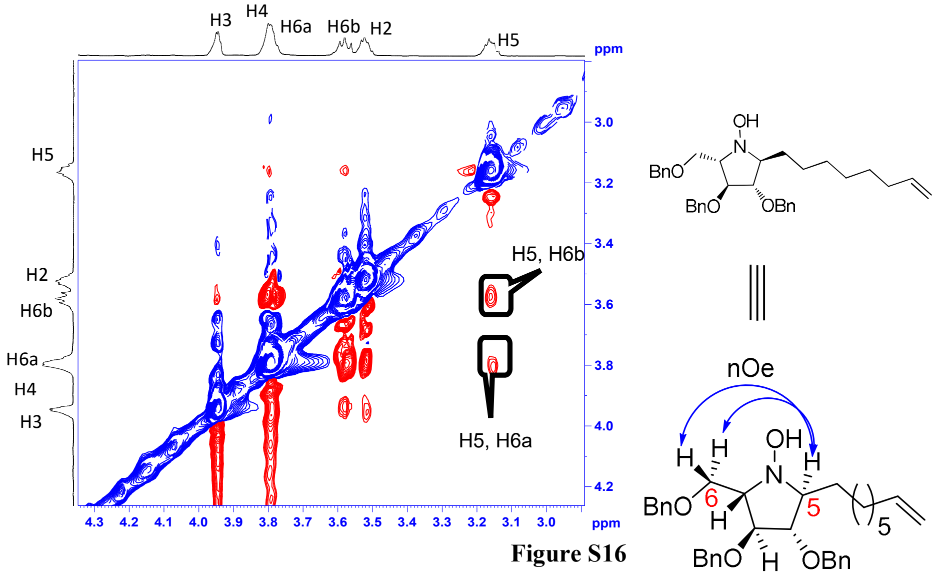

Figure S16

Compound *ent*-3-*epi*-19

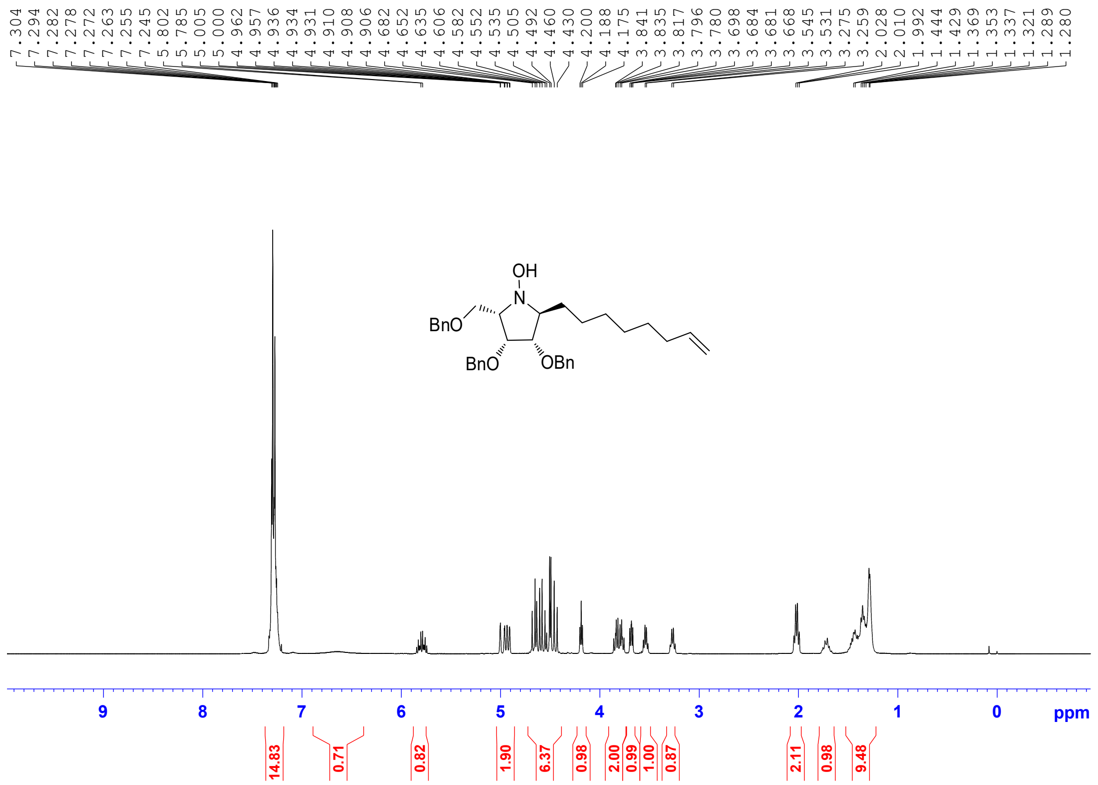

Figure S17

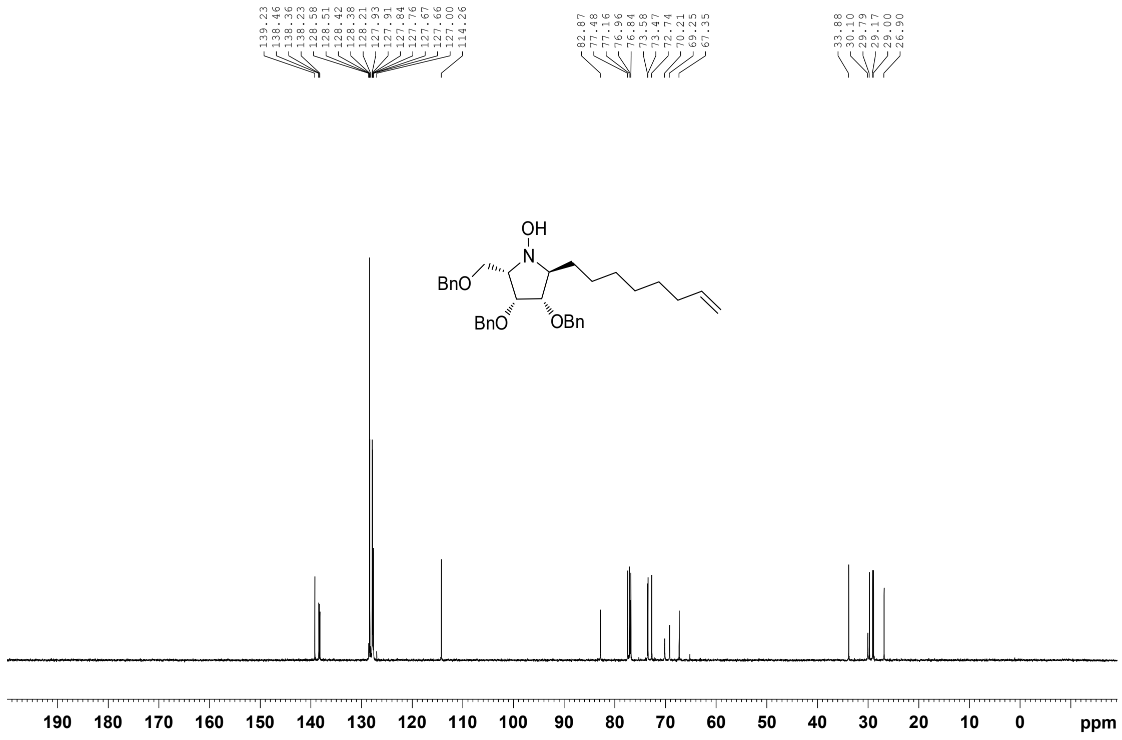

Figure S18

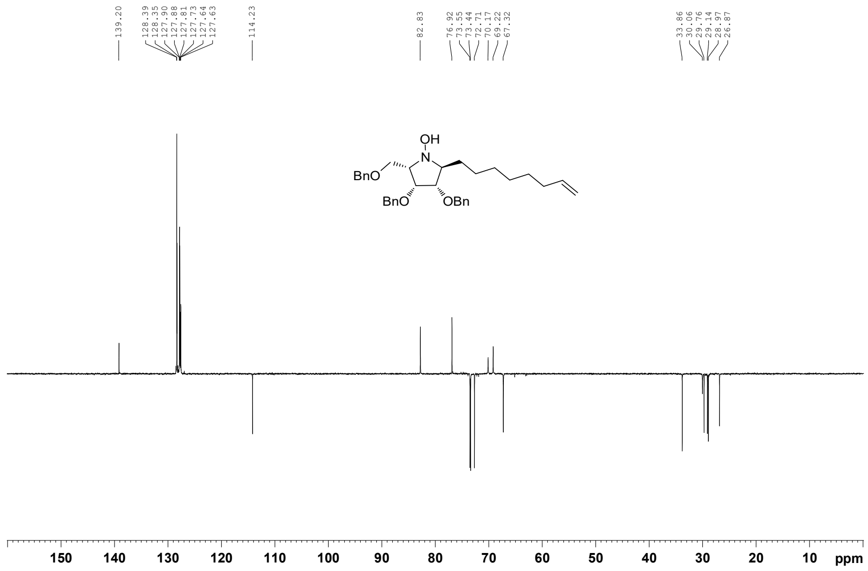

Figure S19

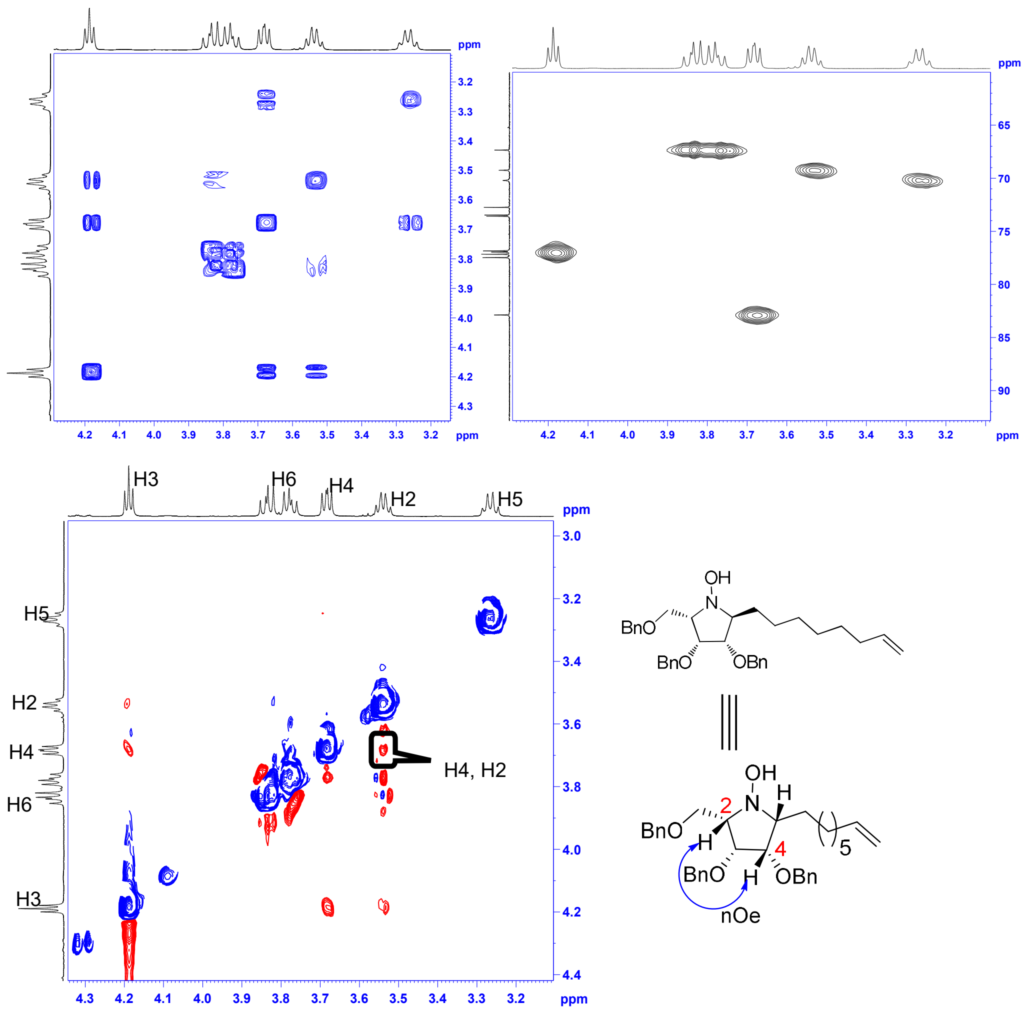

Figure S20

Compound 2-*epi*-19

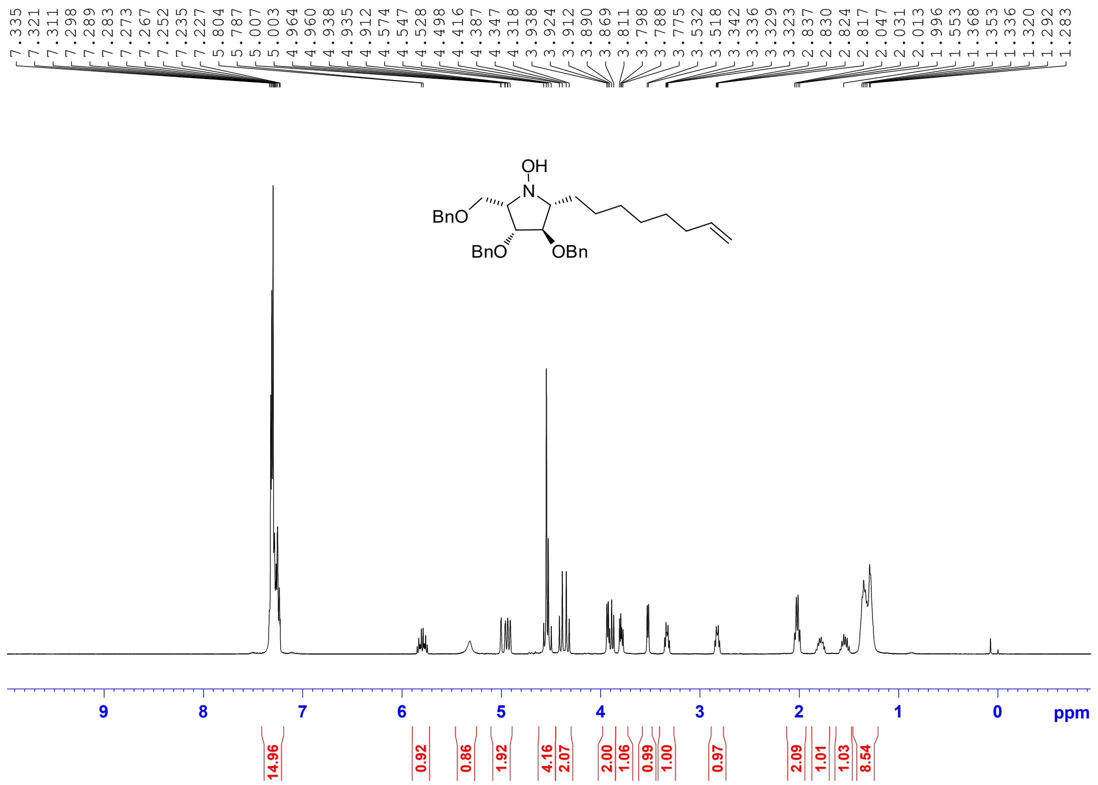

Figure S21

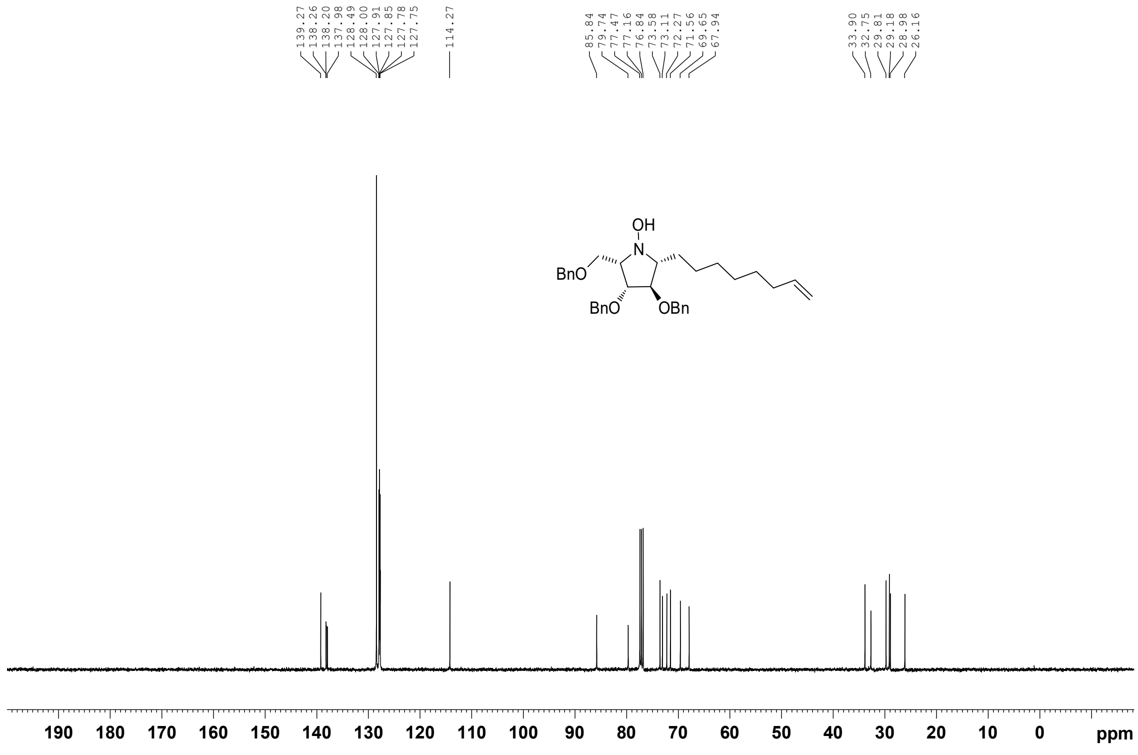

Figure S22

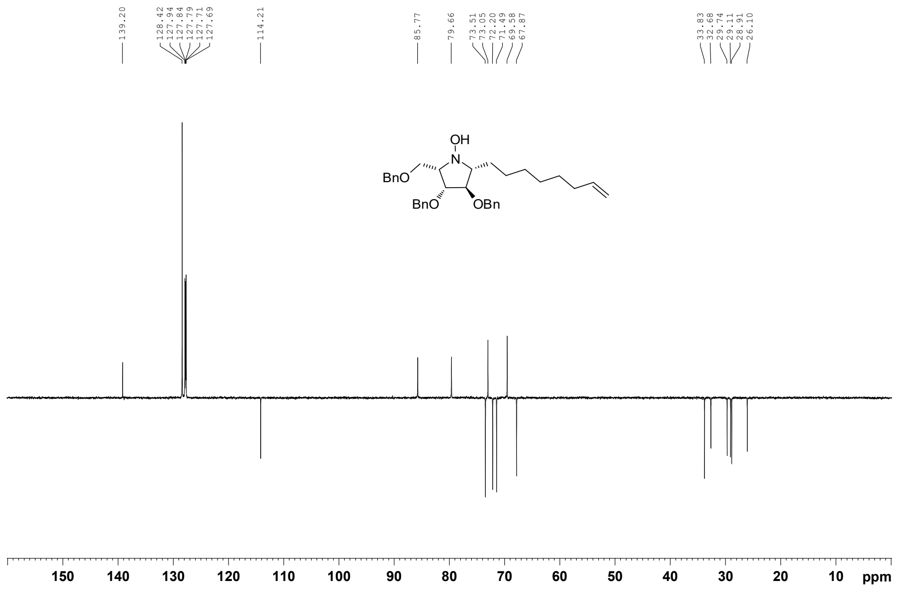

Figure S23

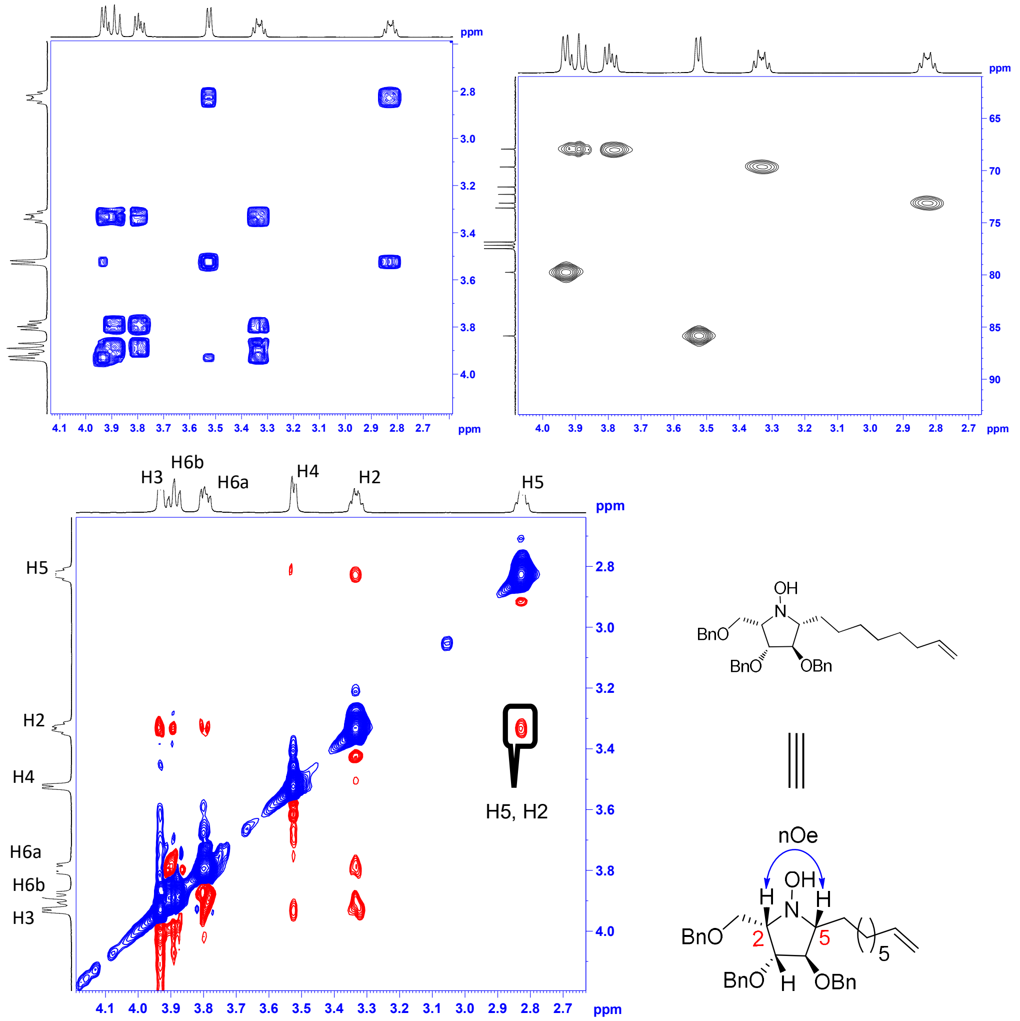

Figure S24

Compound 13

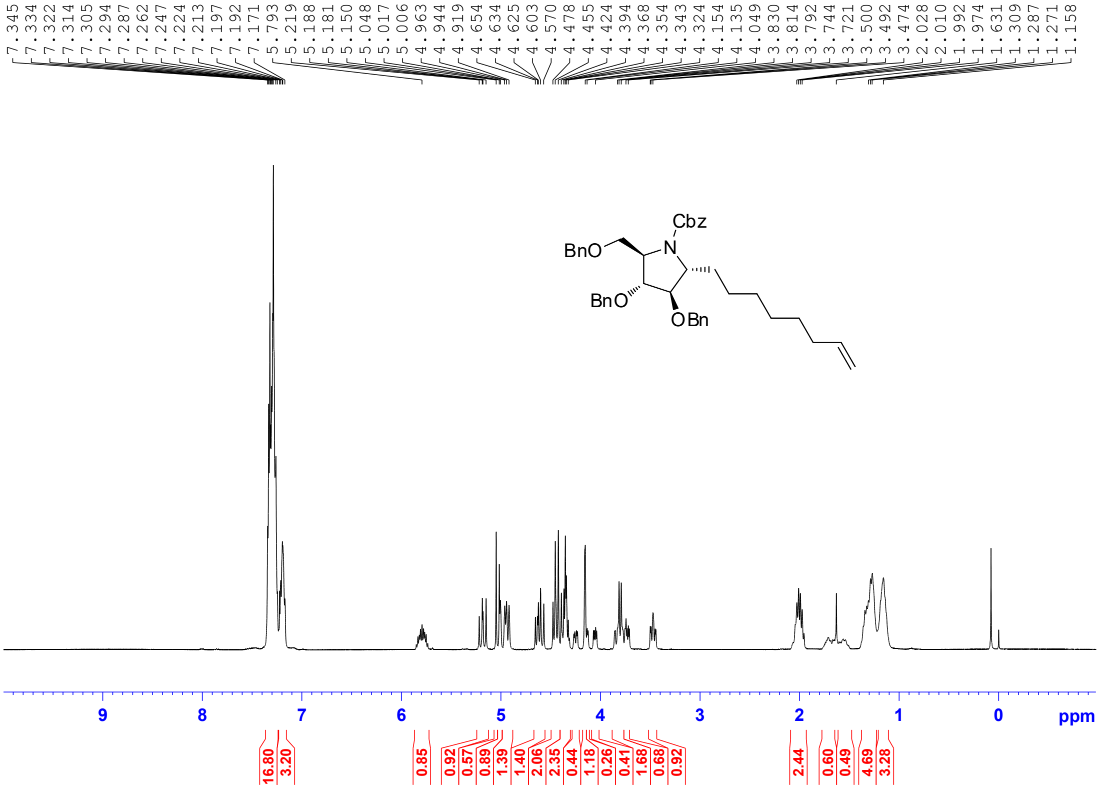

Figure S25

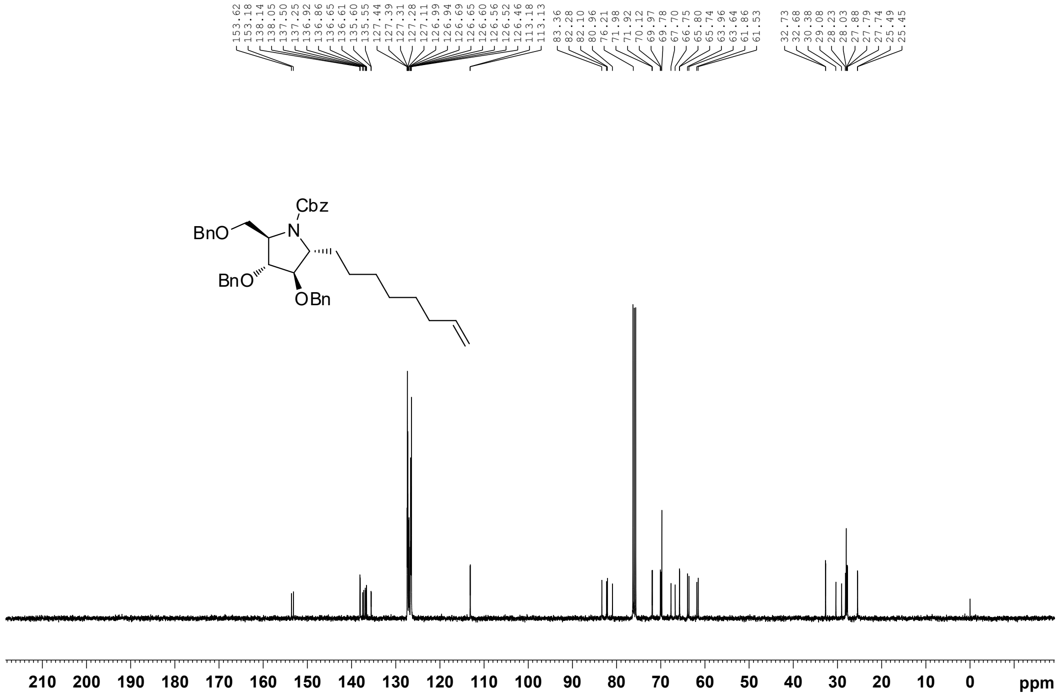

Figure S26

Compound *ent*-13

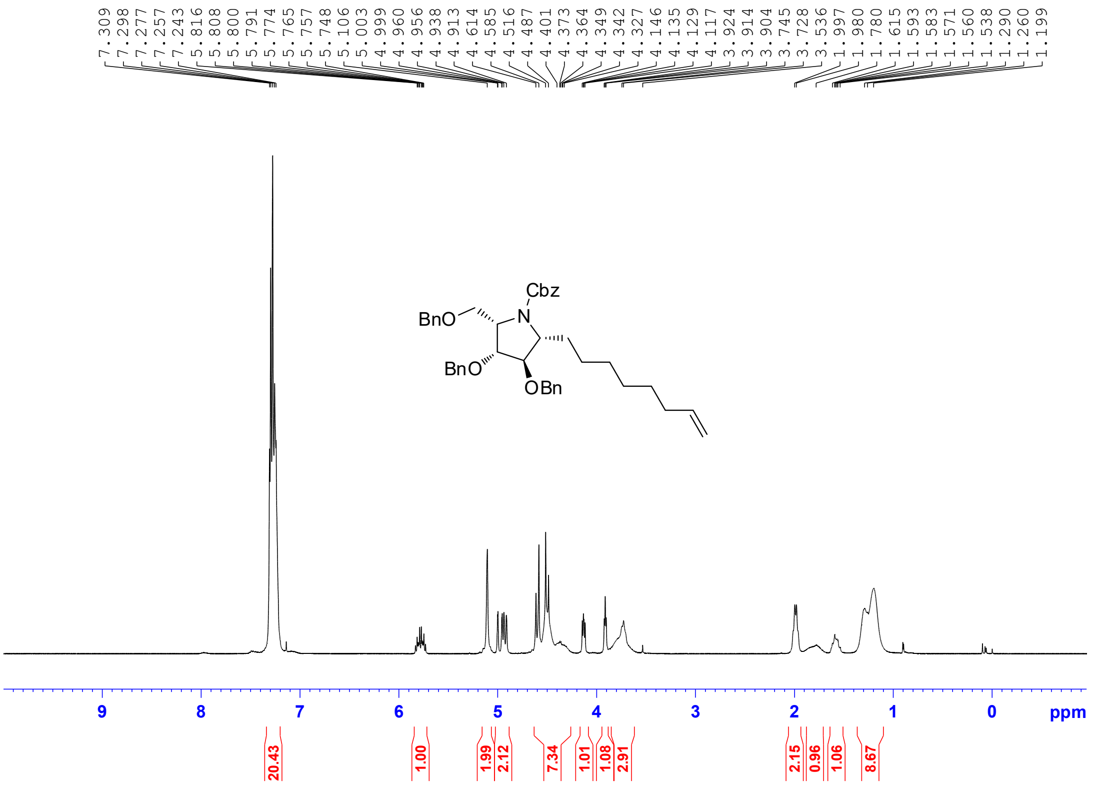

Figure S27

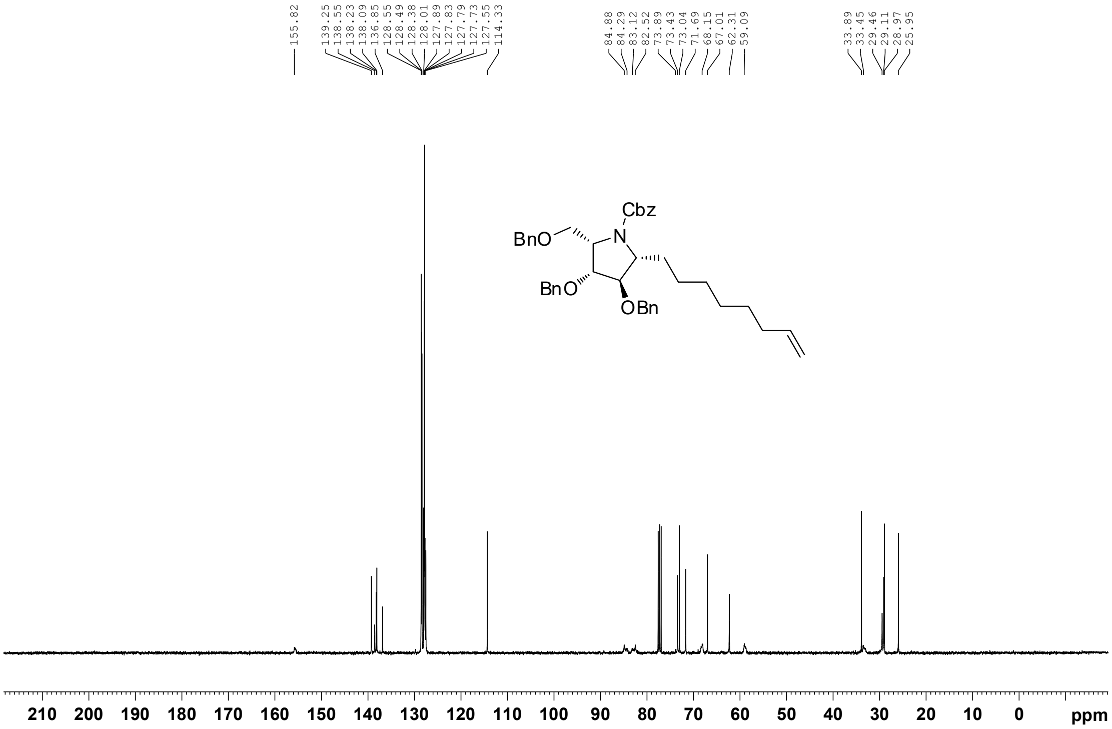

Figure S28

Compound *ent*-3-*epi*-13

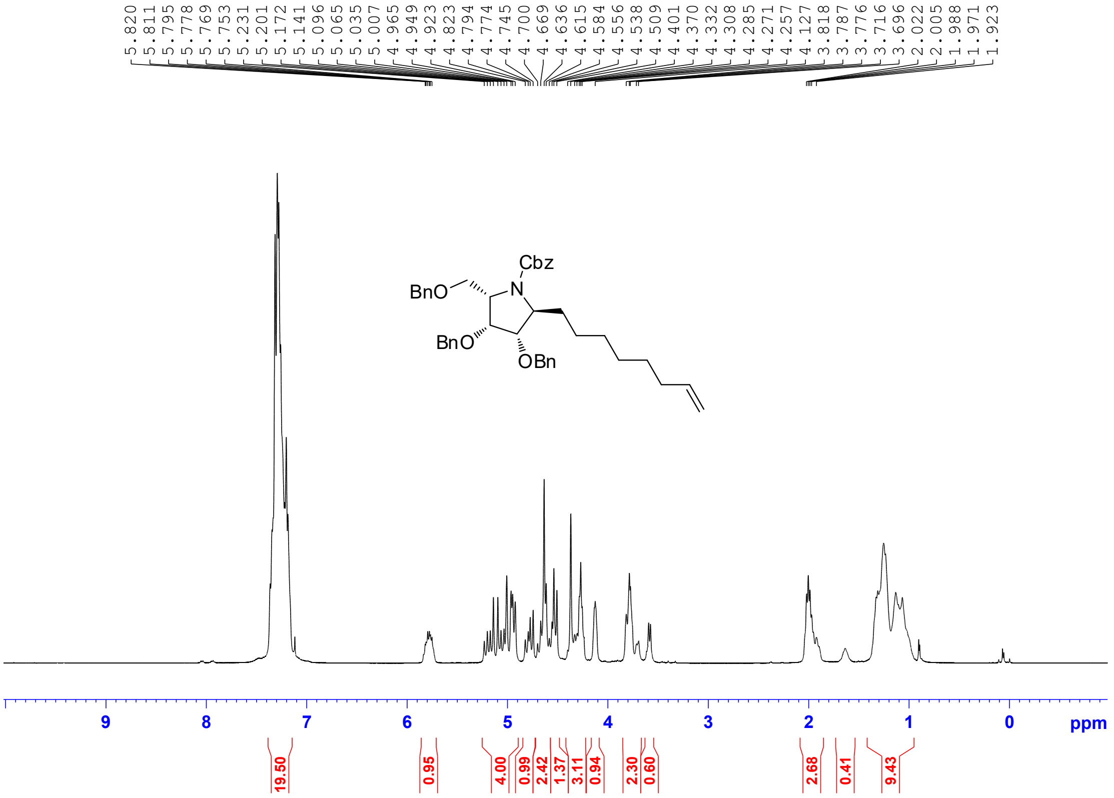

Figure S29

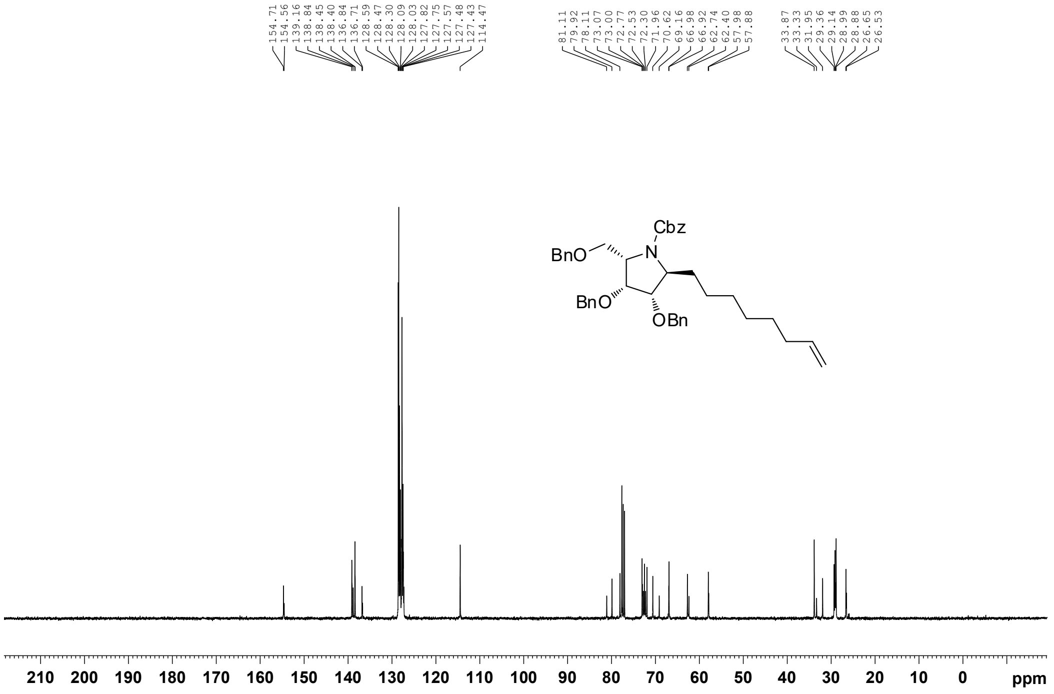

Figure S30

Compound 2-*epi*-13

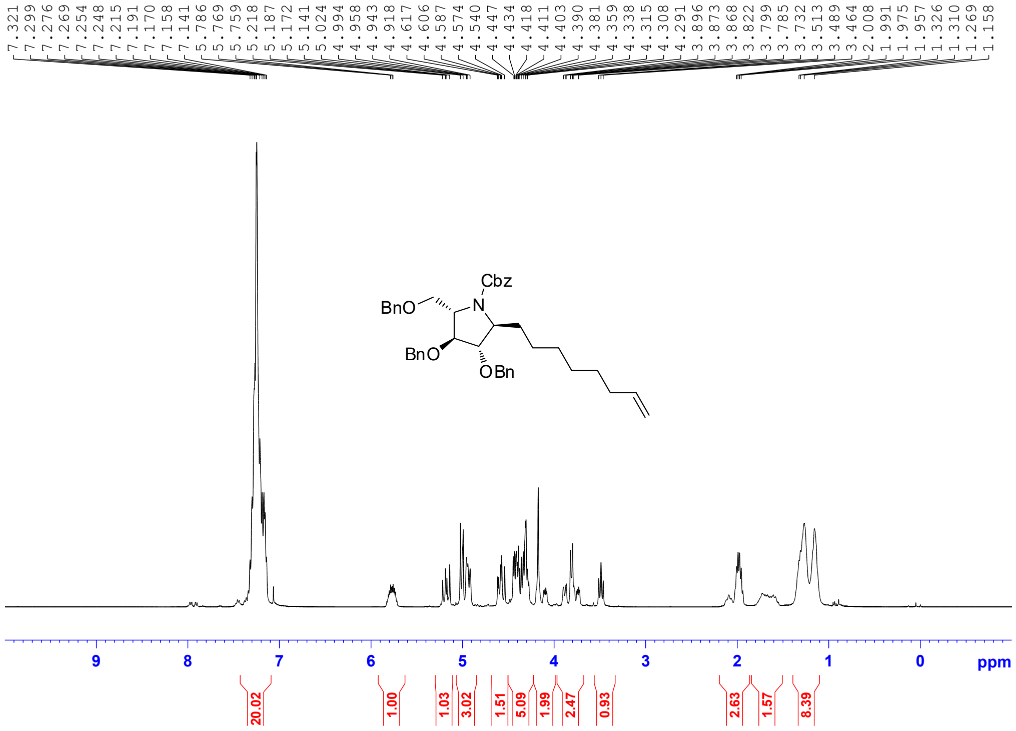

Figure S31

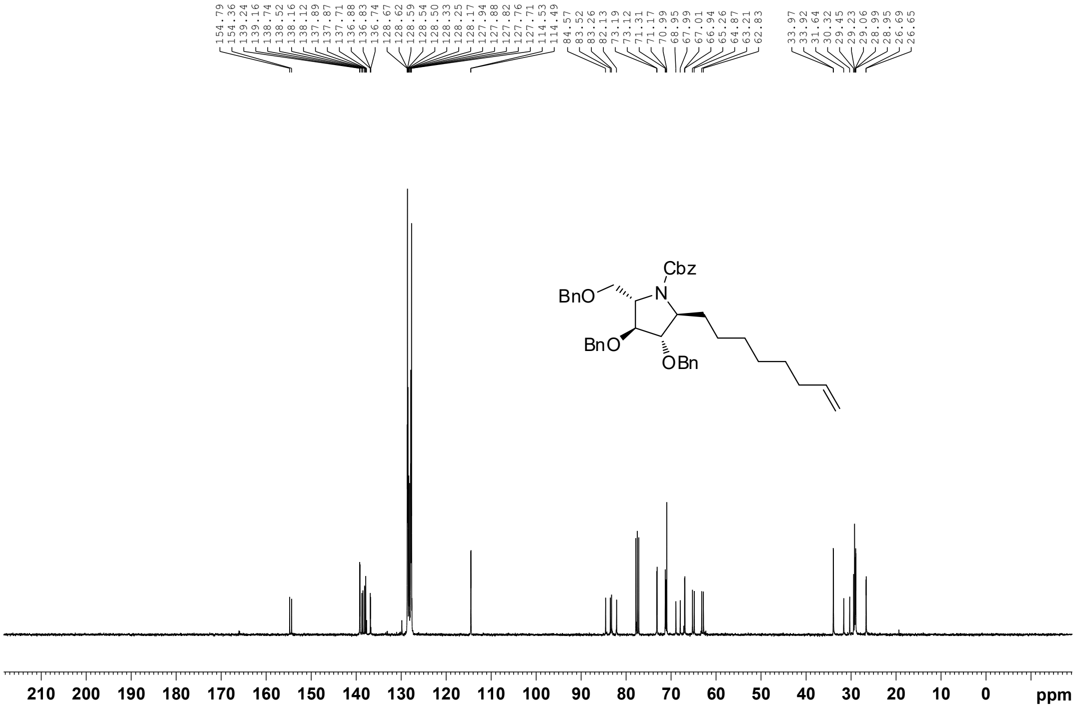

Figure S32

Compound 12

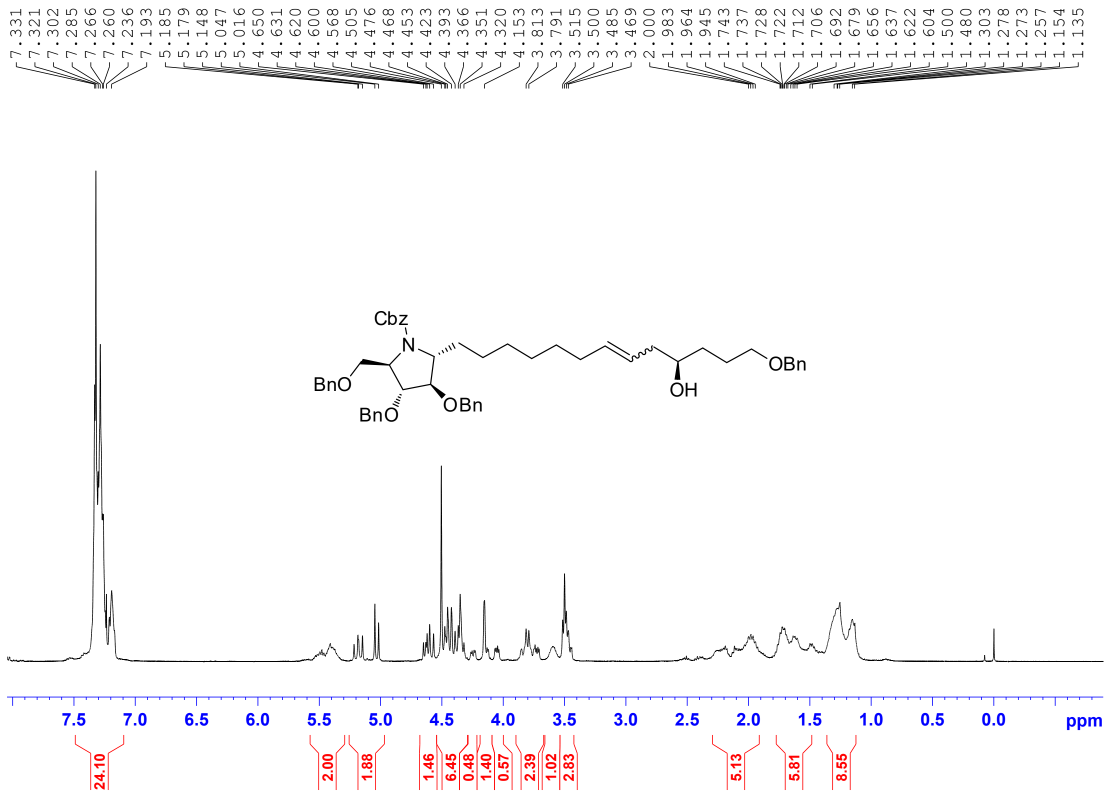

Figure S33

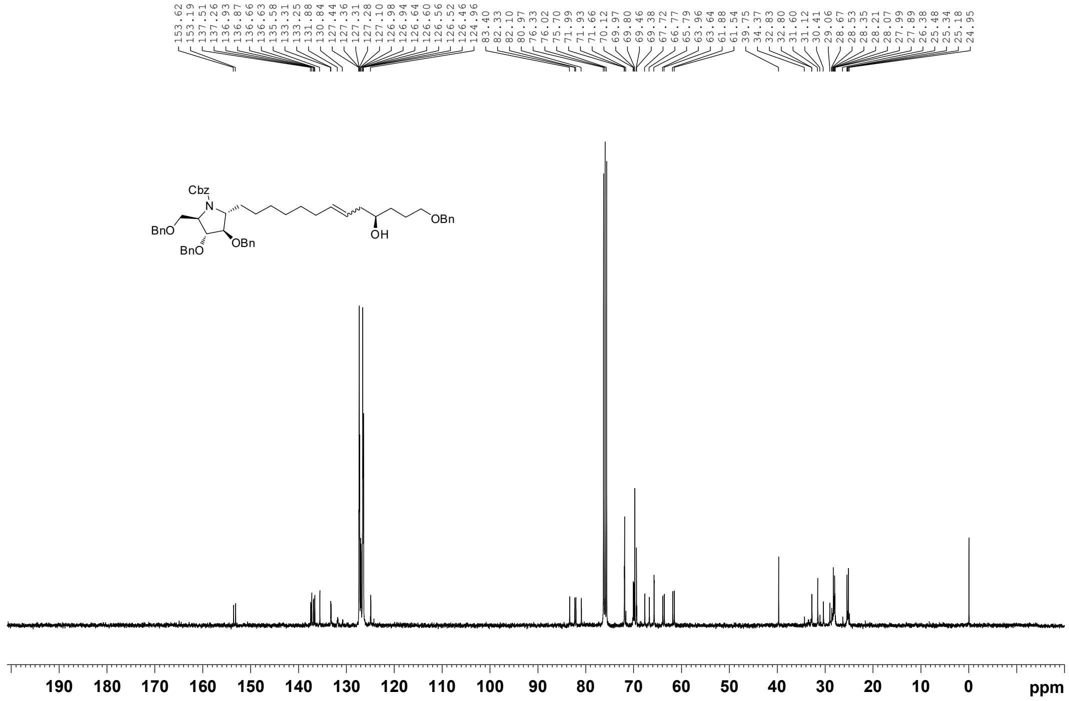

Figure S34

Compound 10'-*epi*-12

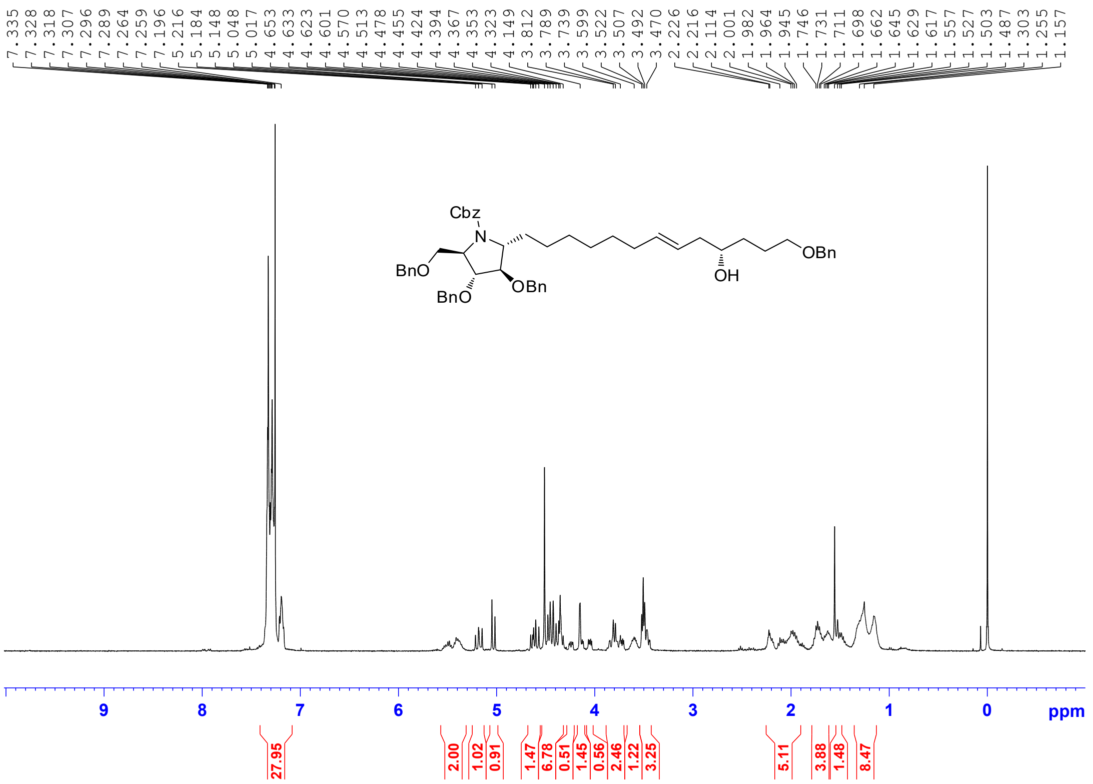

Figure S35

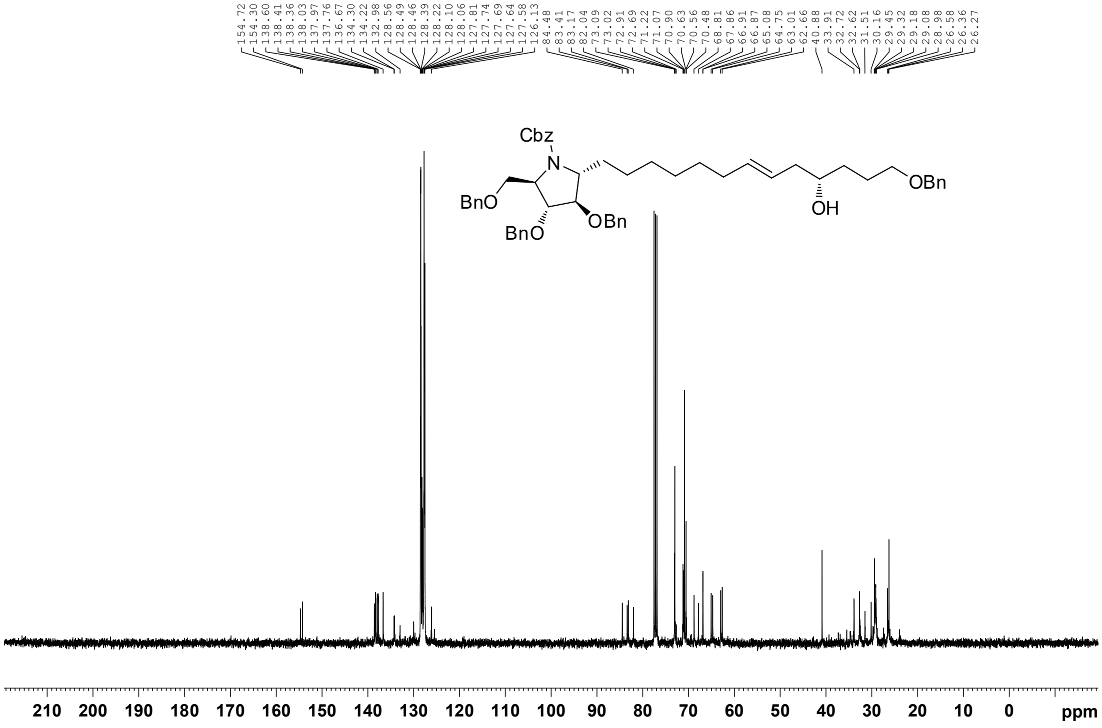

Figure S36

Compound *ent*-10'-*epi*-12

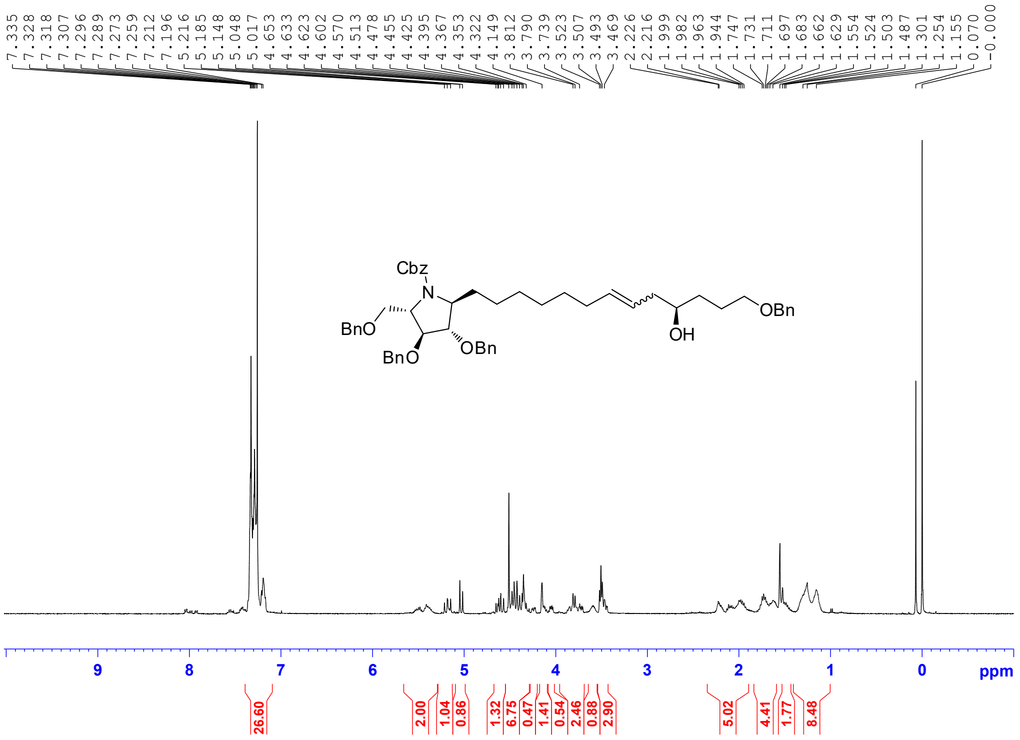

Figure S37

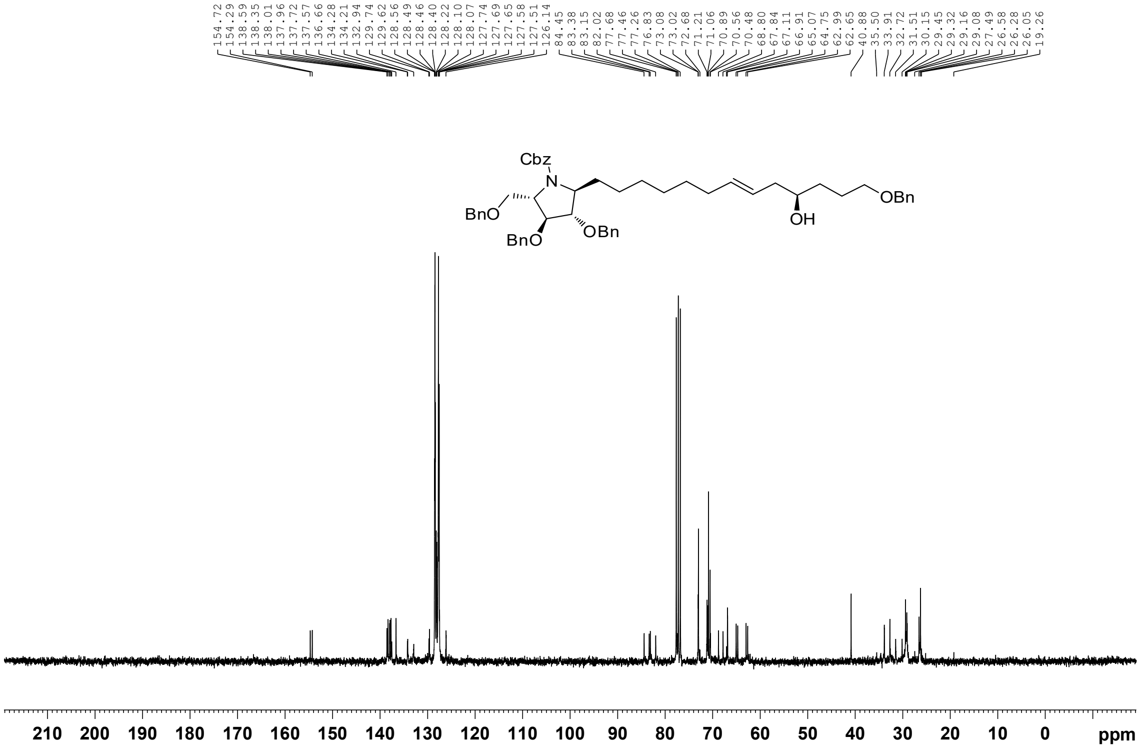

Figure S38

Compound *ent*-12

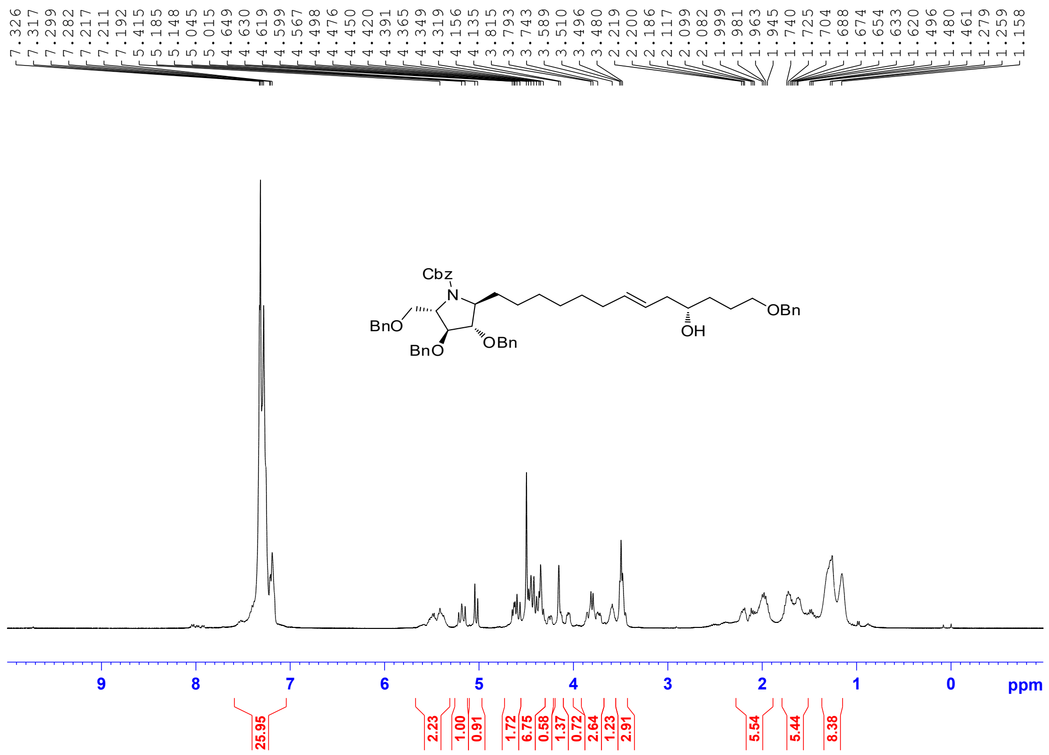

Figure S39

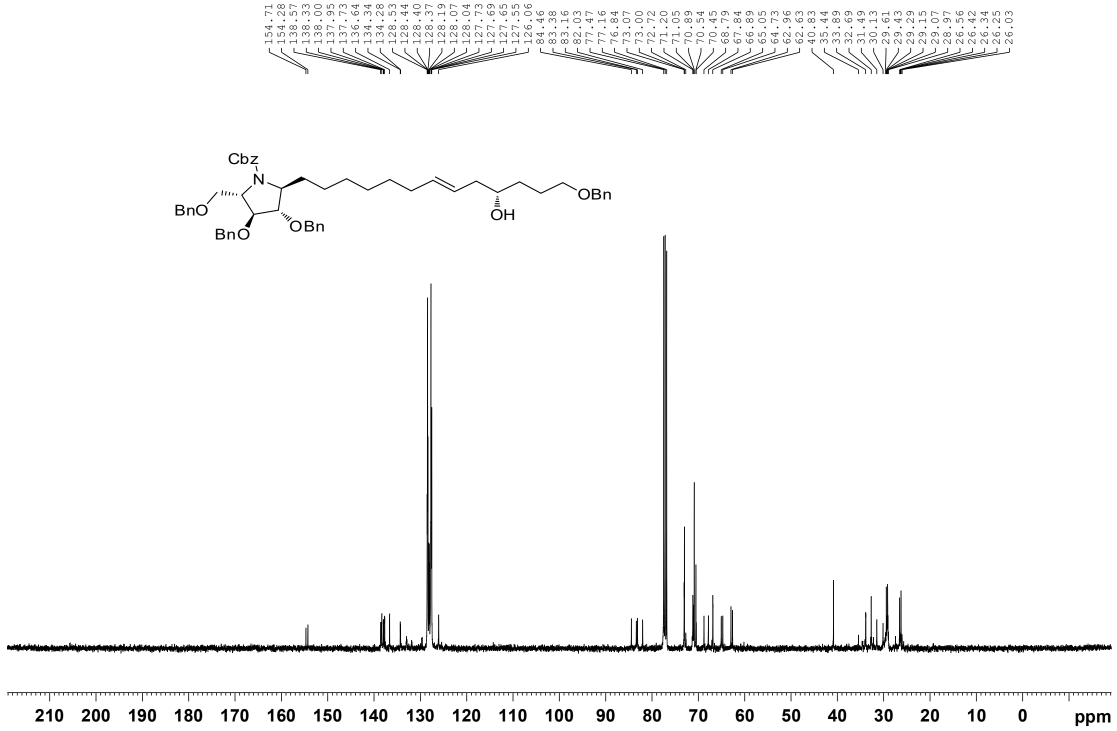

Figure S40

Compound *ent*-3,10’-di-*epi*-12

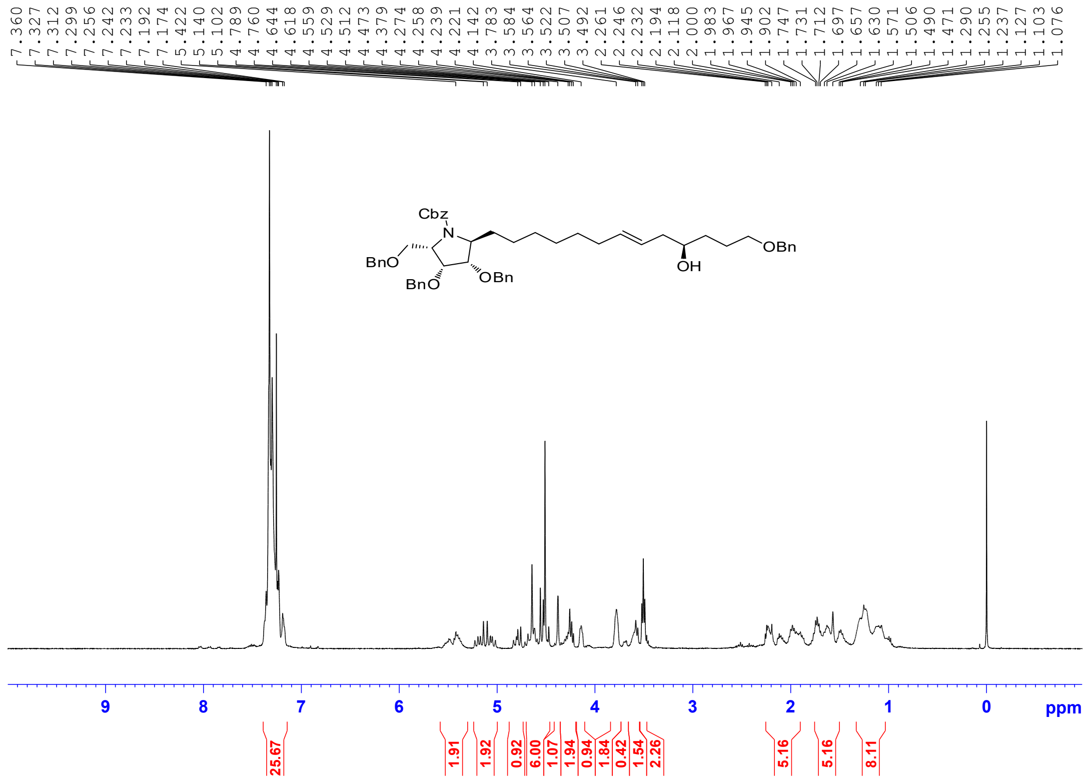

Figure S41

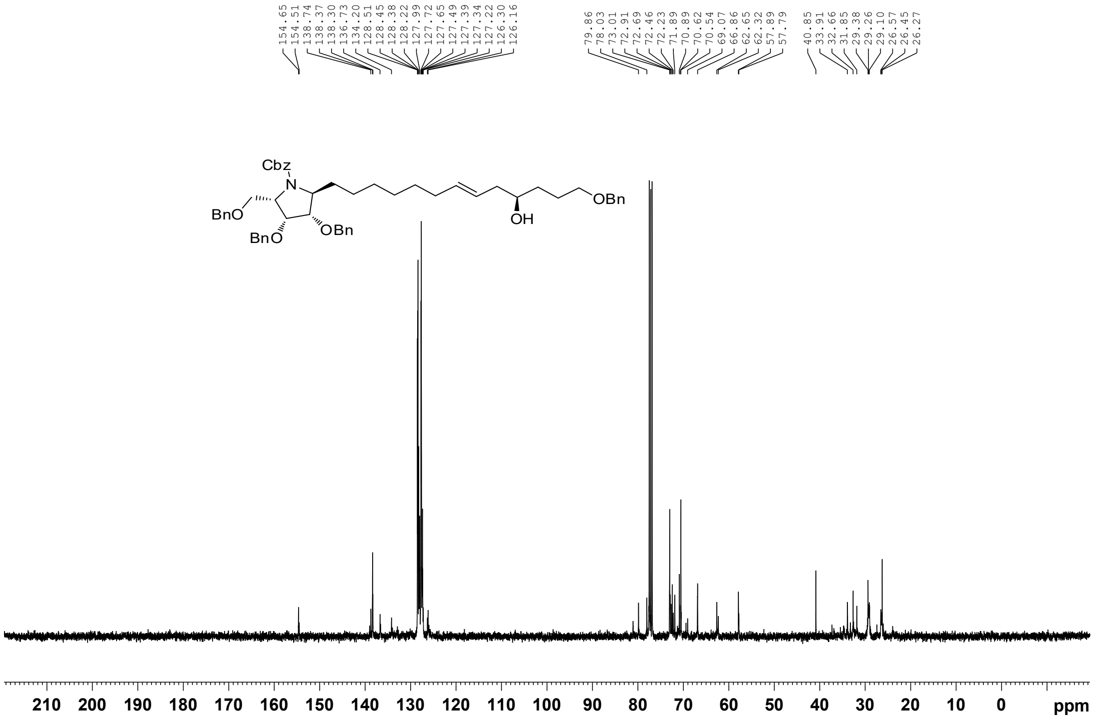

Figure S42

Compound *ent*-3-*epi*-12

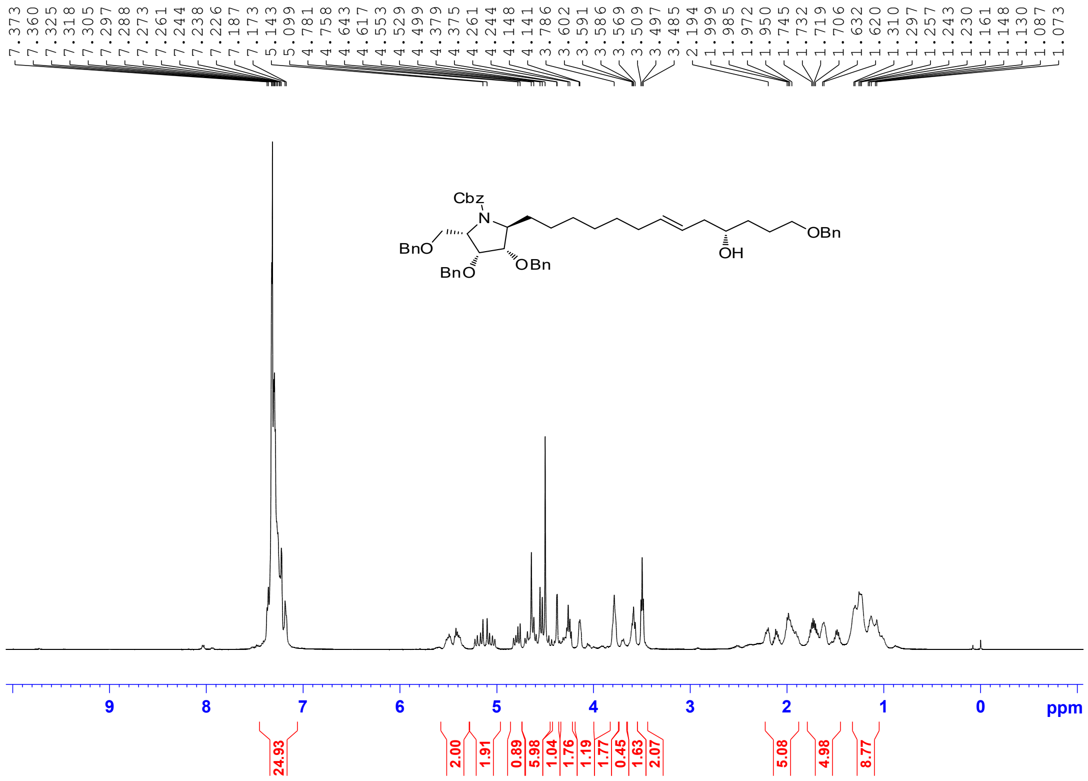

Figure S43

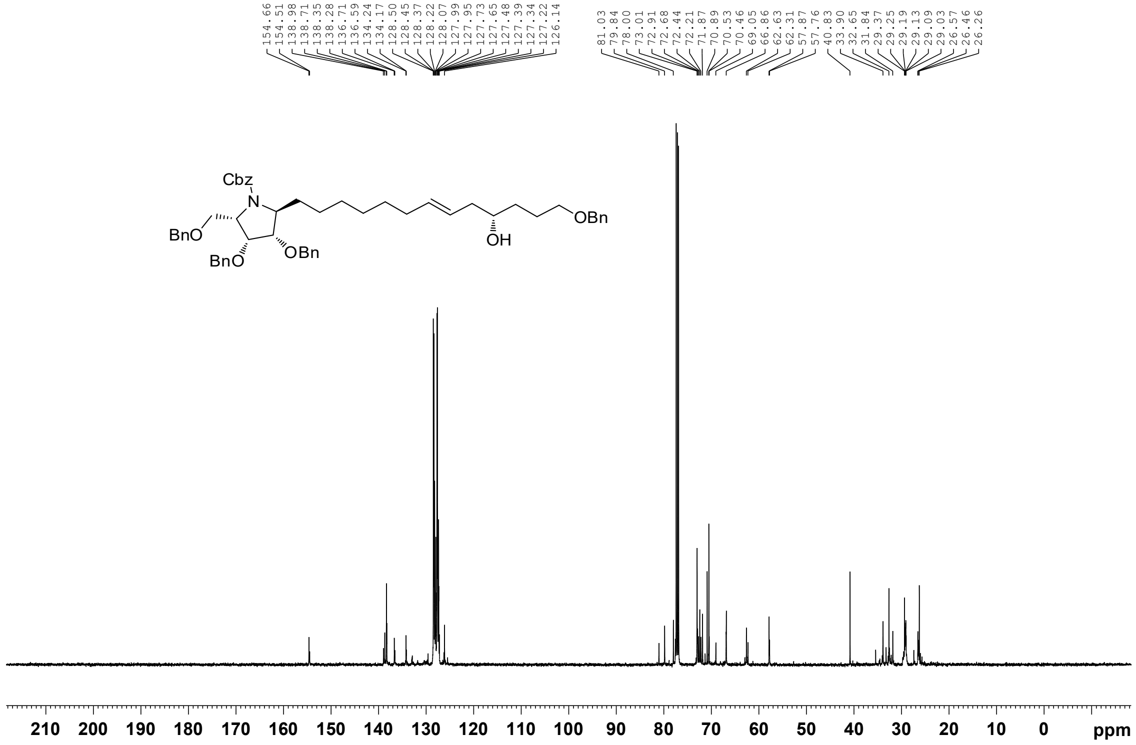

Figure S44

Compound 2-*epi*-12

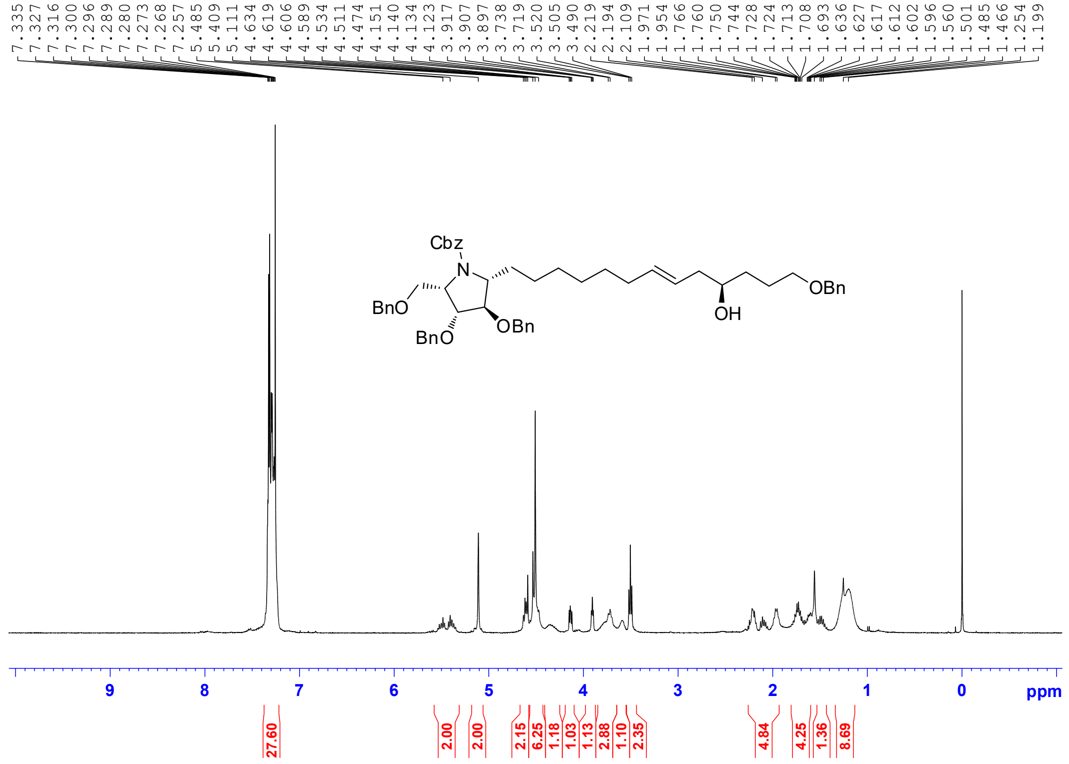

Figure S45

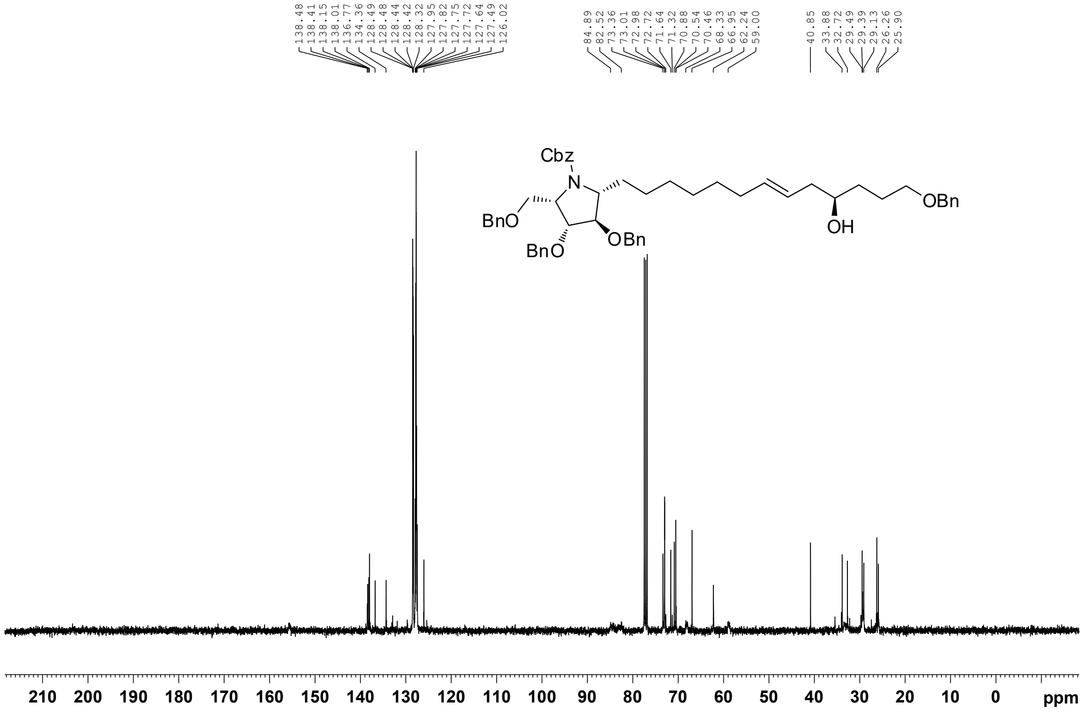

Figure S46

Compound 2,10'-di-*epi*-12

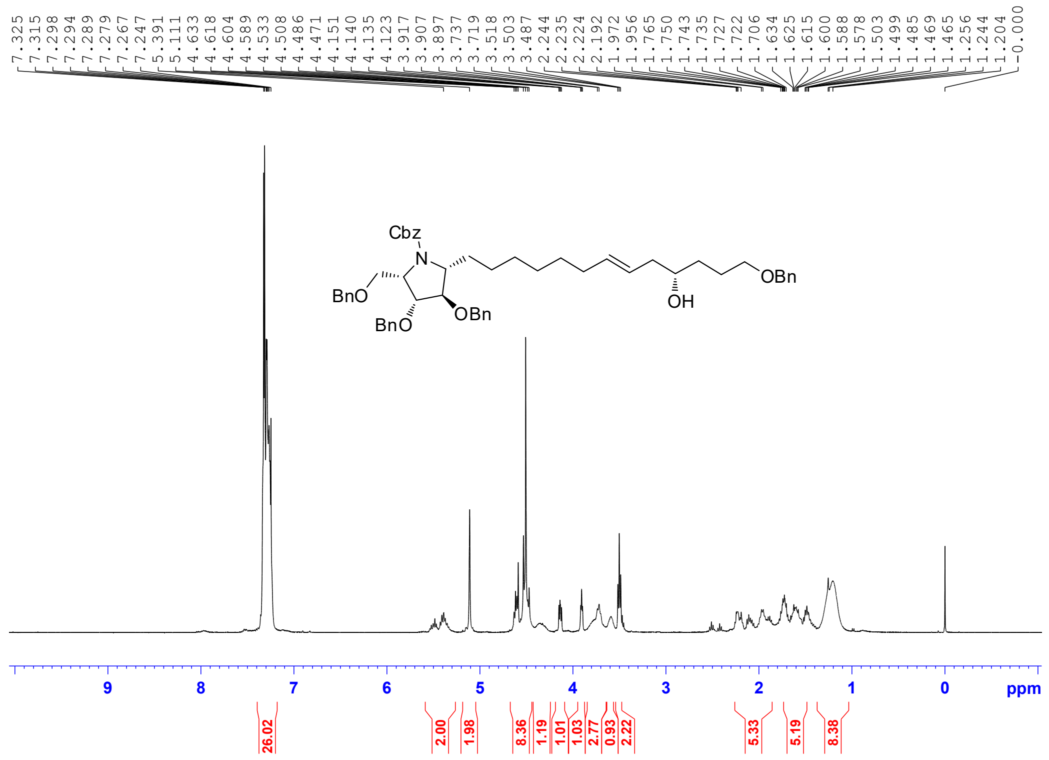

Figure S47

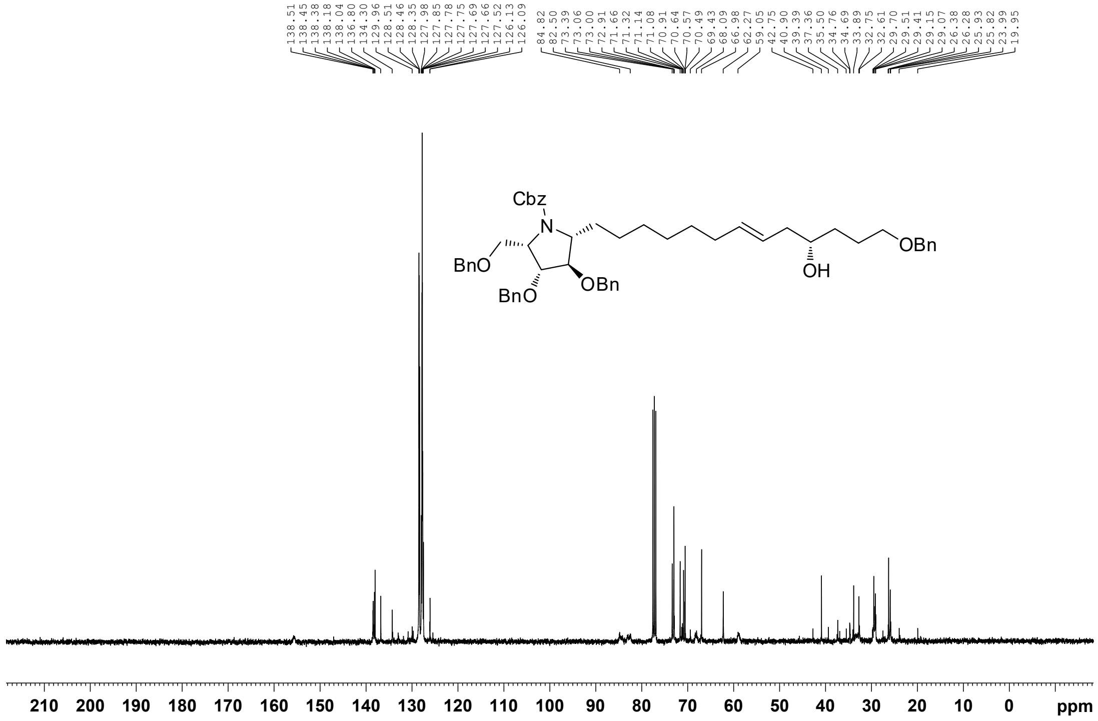

Figure S48

Compound 3 (CD_3OD)

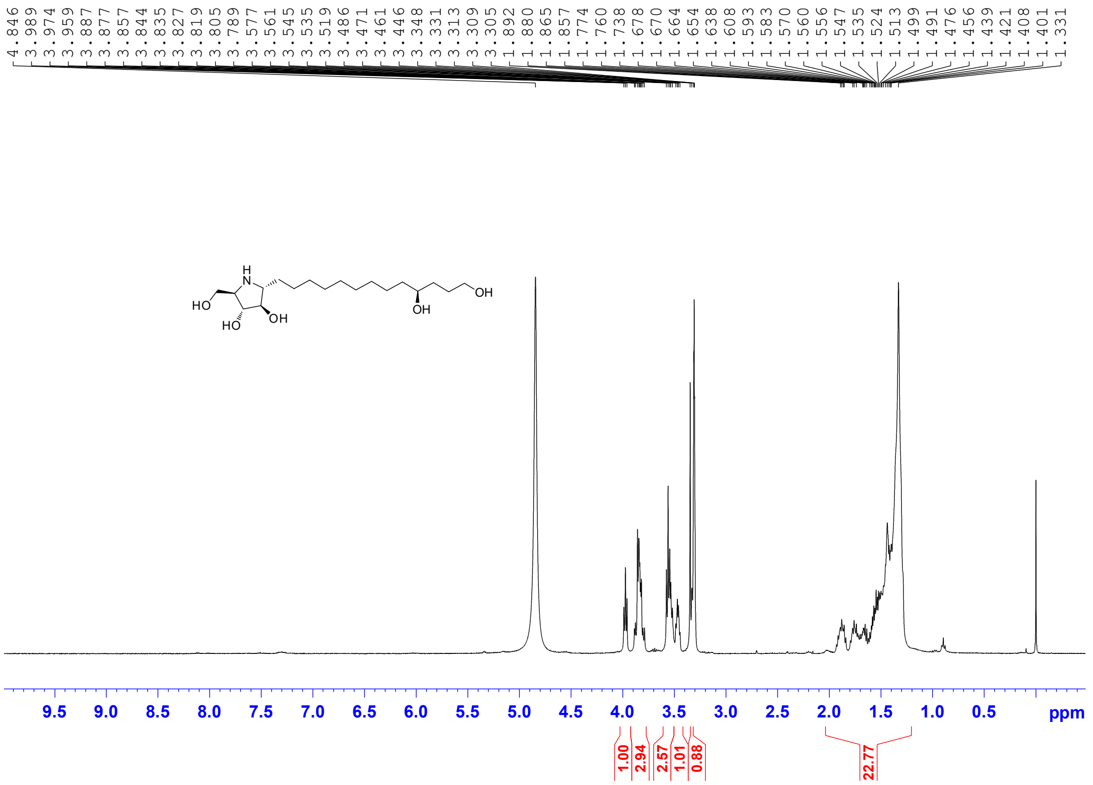

Figure S49

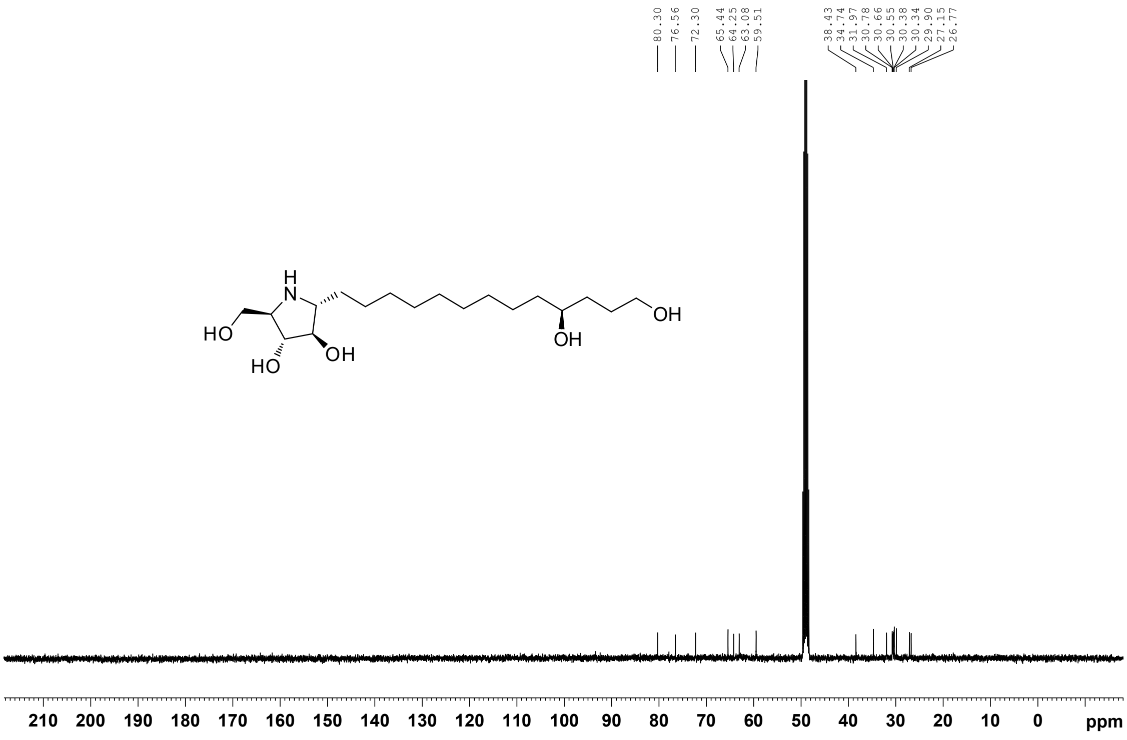

Figure S50

Compound 3 (C_5D_5N)

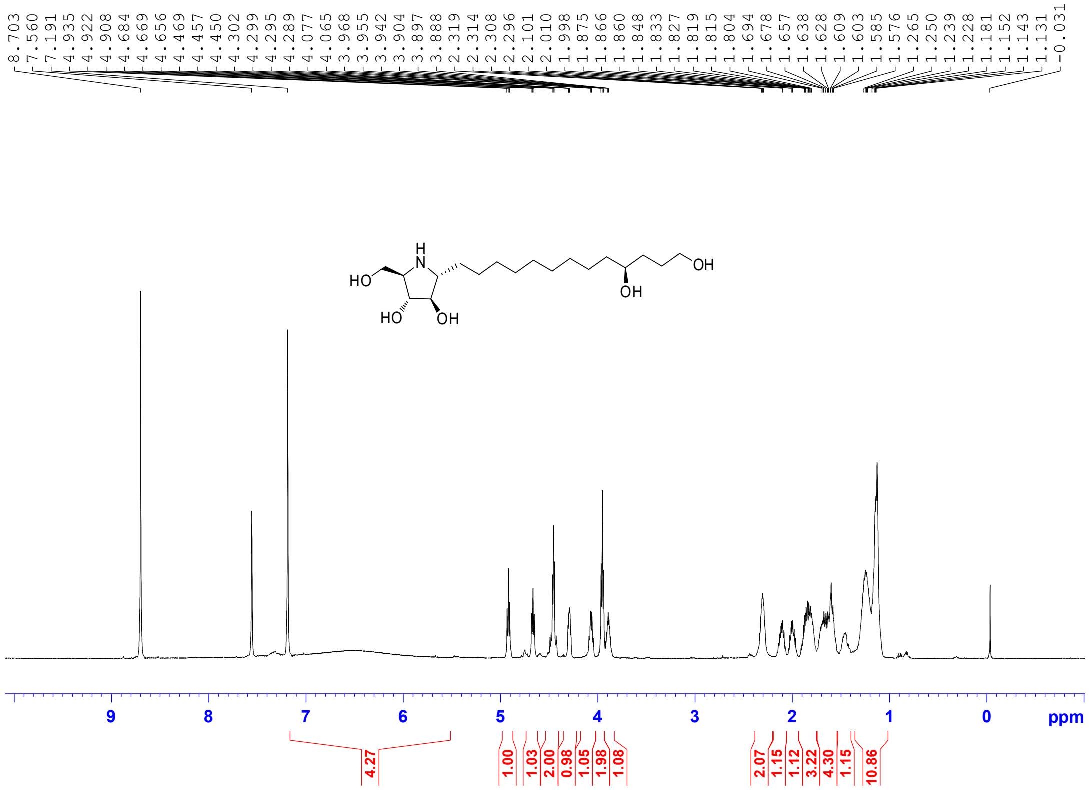

Figure S51

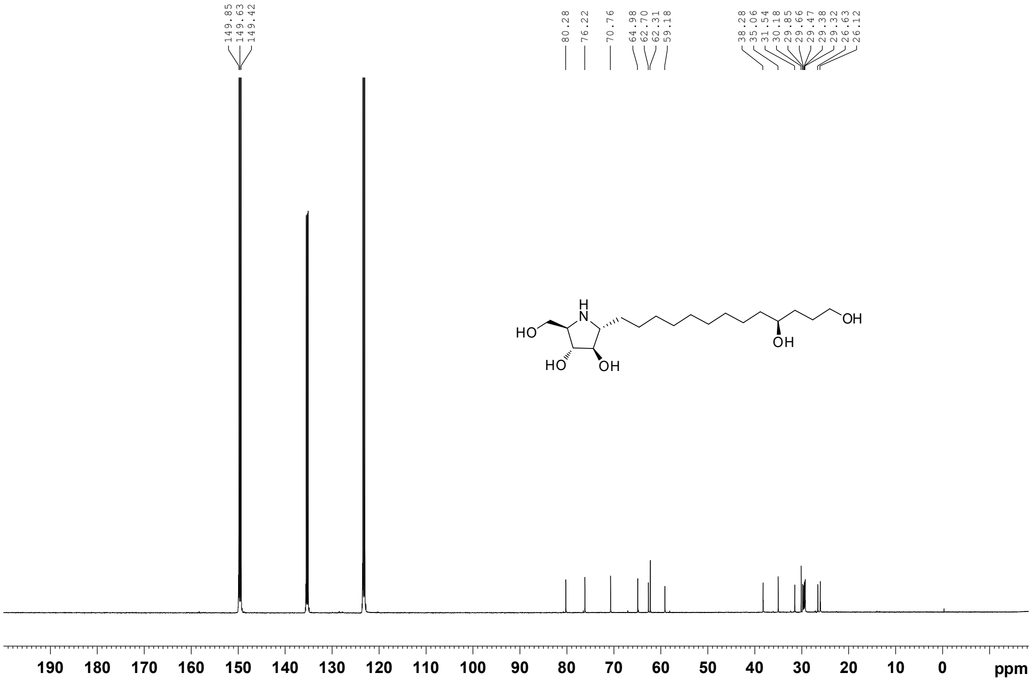

Figure S52

Compound 10'-*epi*-3

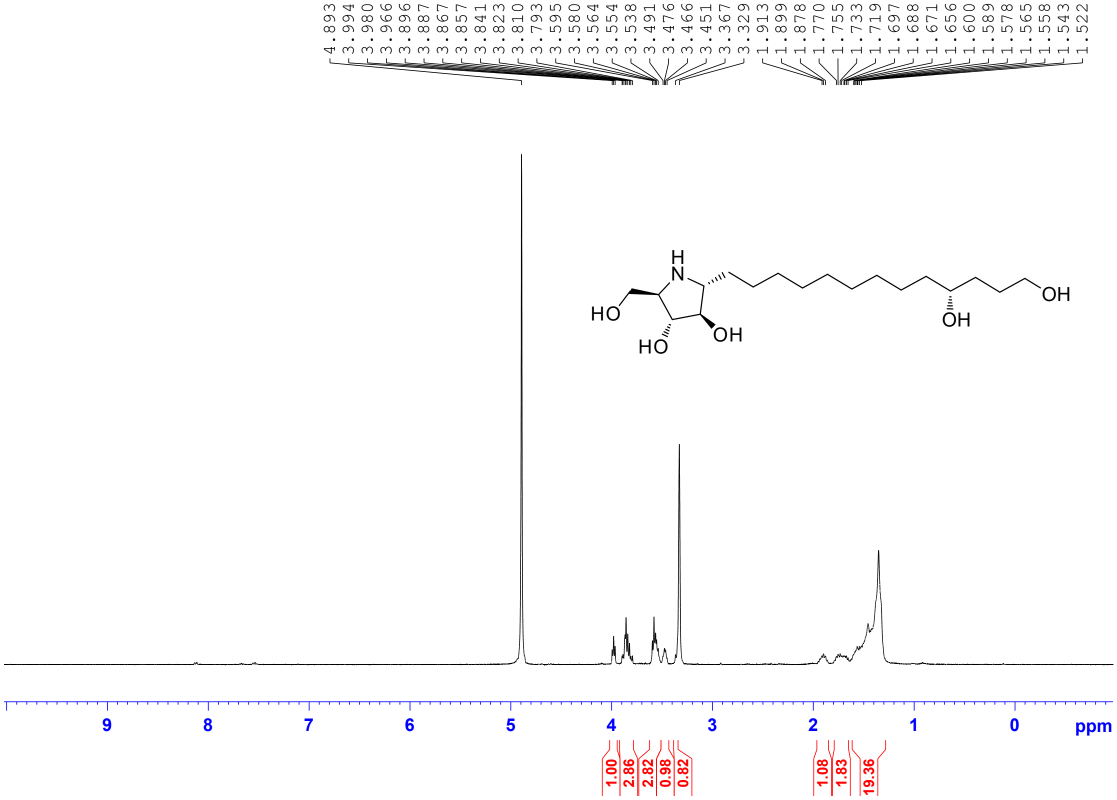

Figure S53

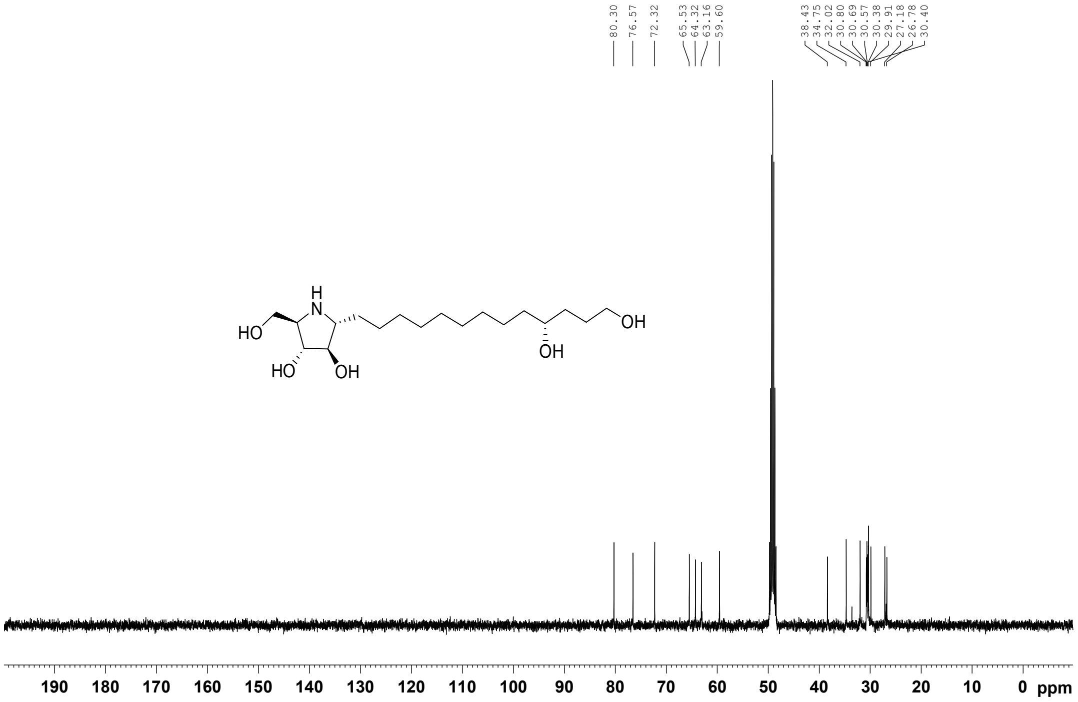

Figure S54

Compound *ent*-10’-*epi*-3

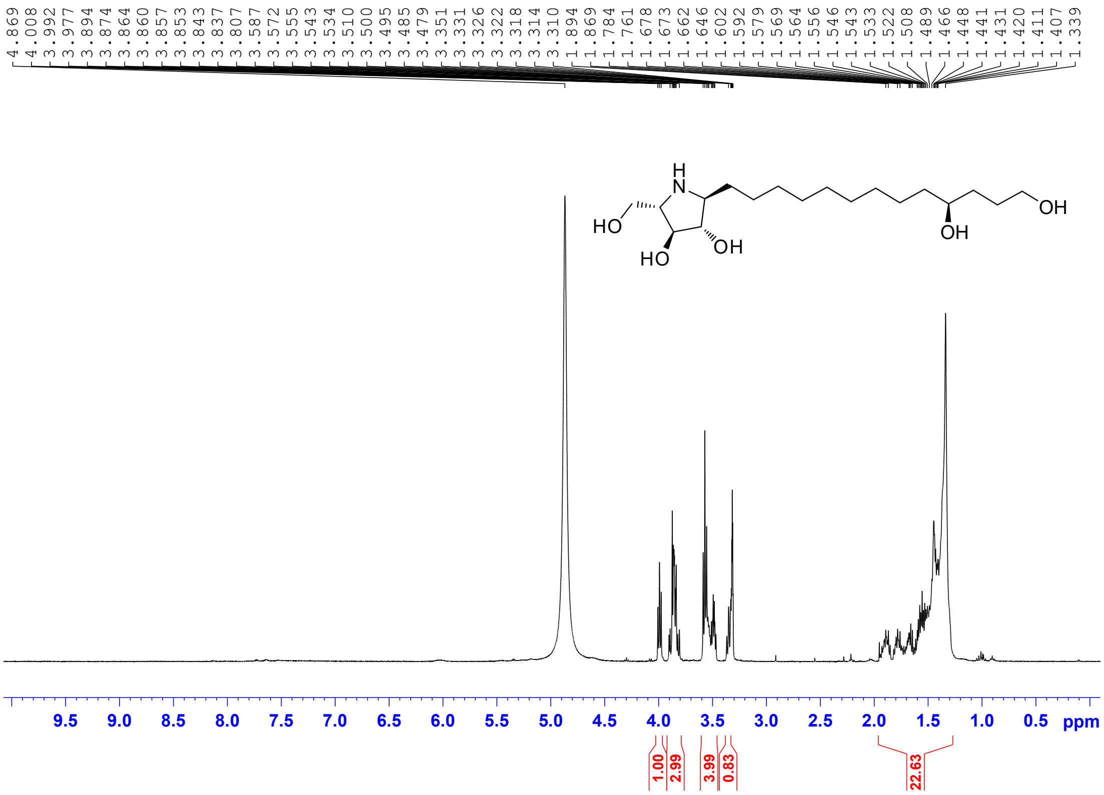

Figure S55

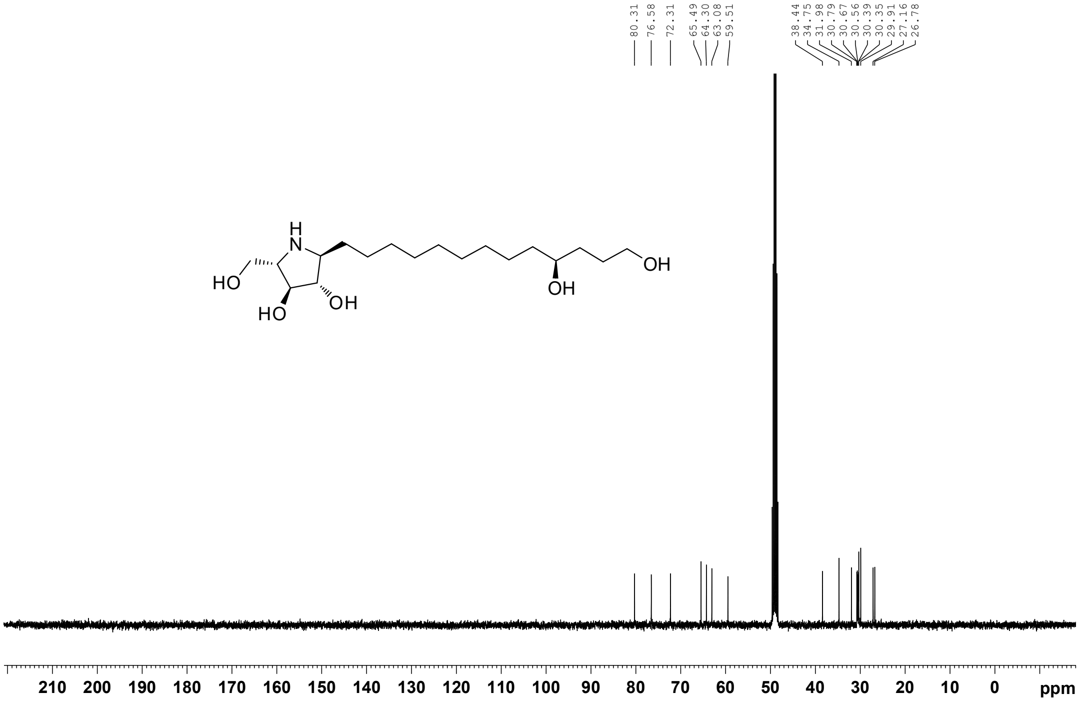

Figure S56

Compound *ent*-3

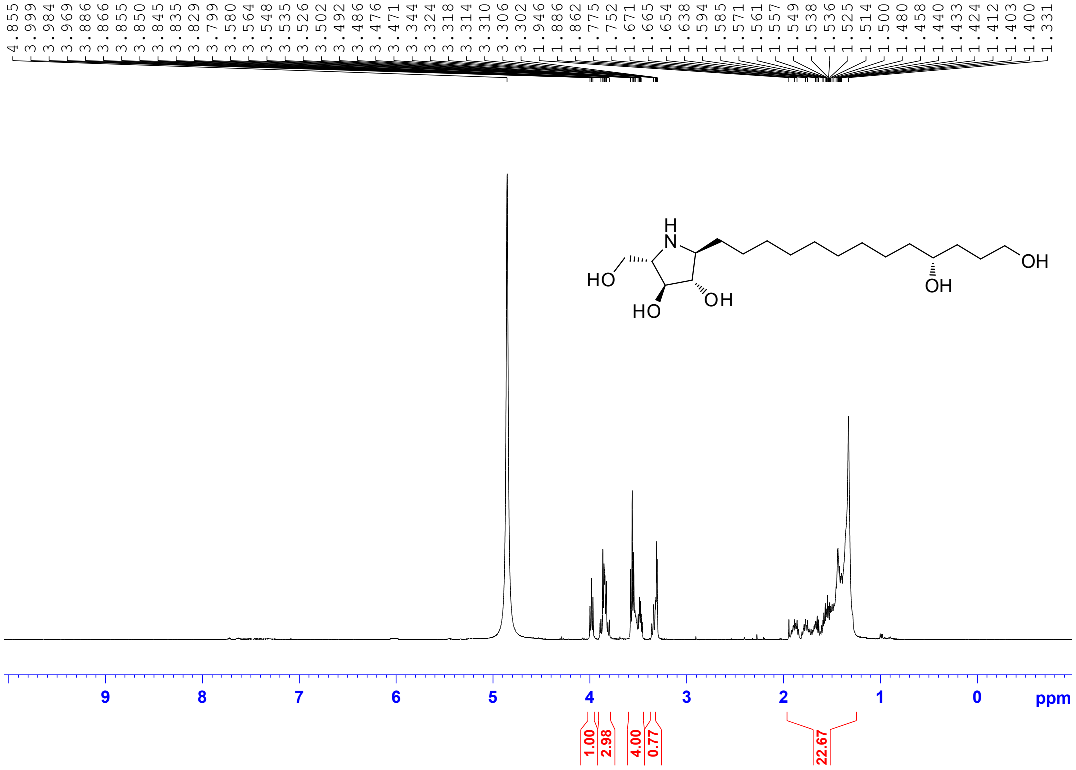

Figure S57

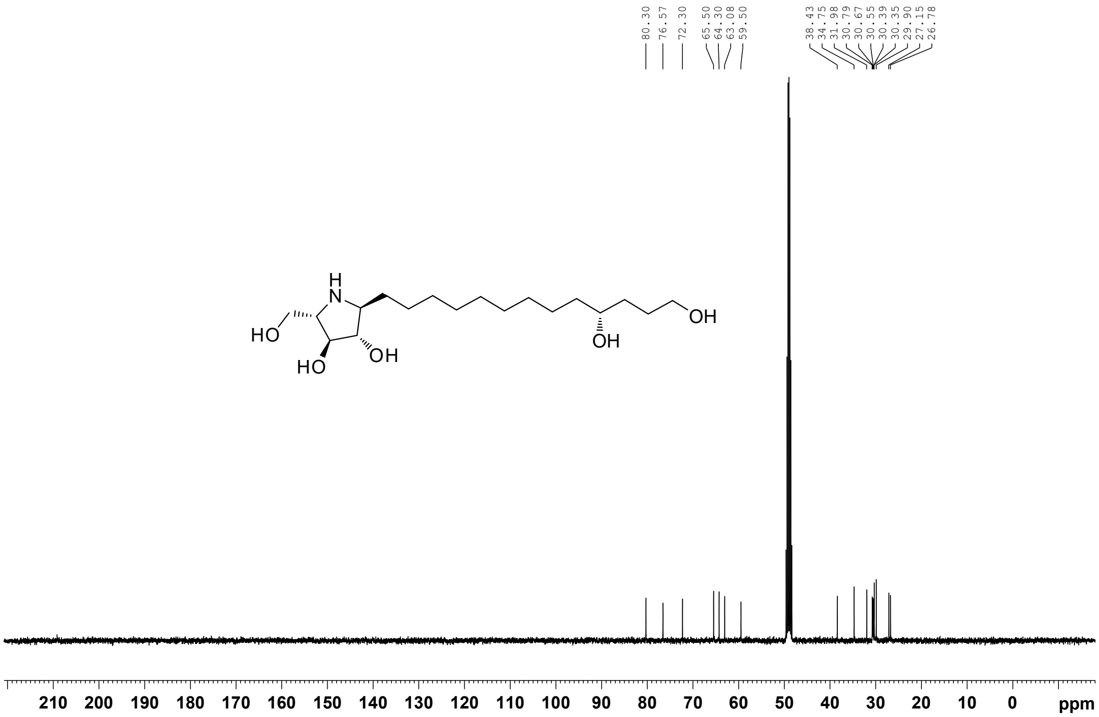

Figure S58

Compound *ent*-3,10'-di-*epi*-3

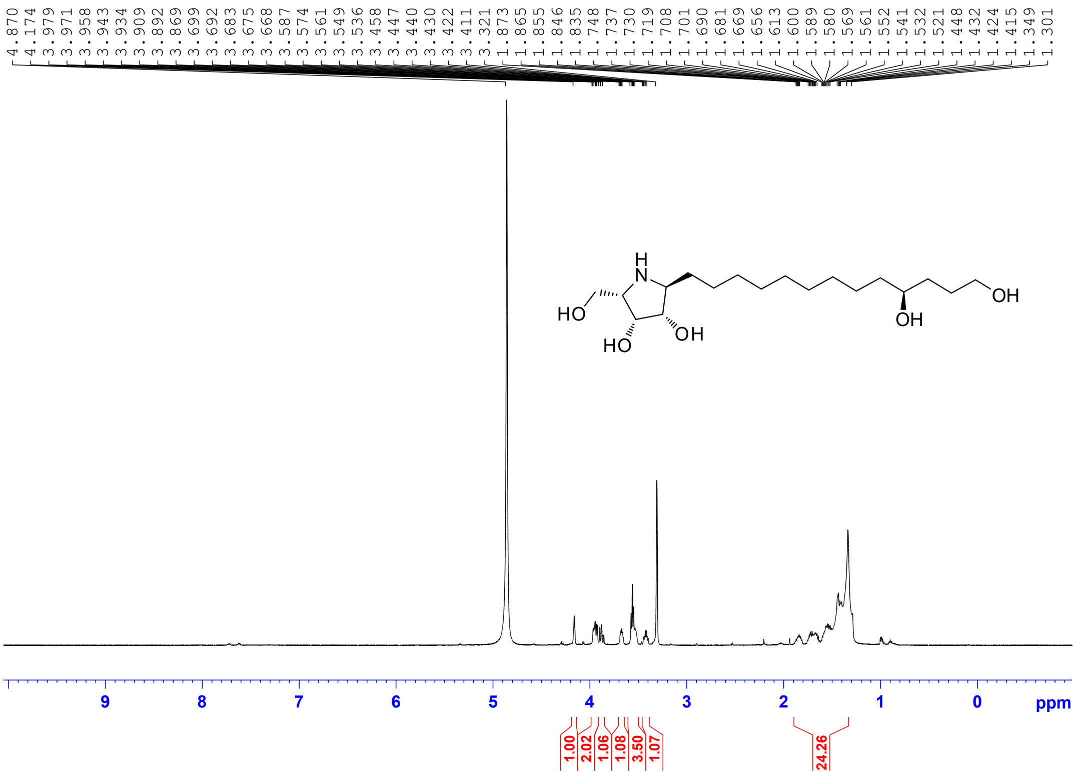

Figure S59

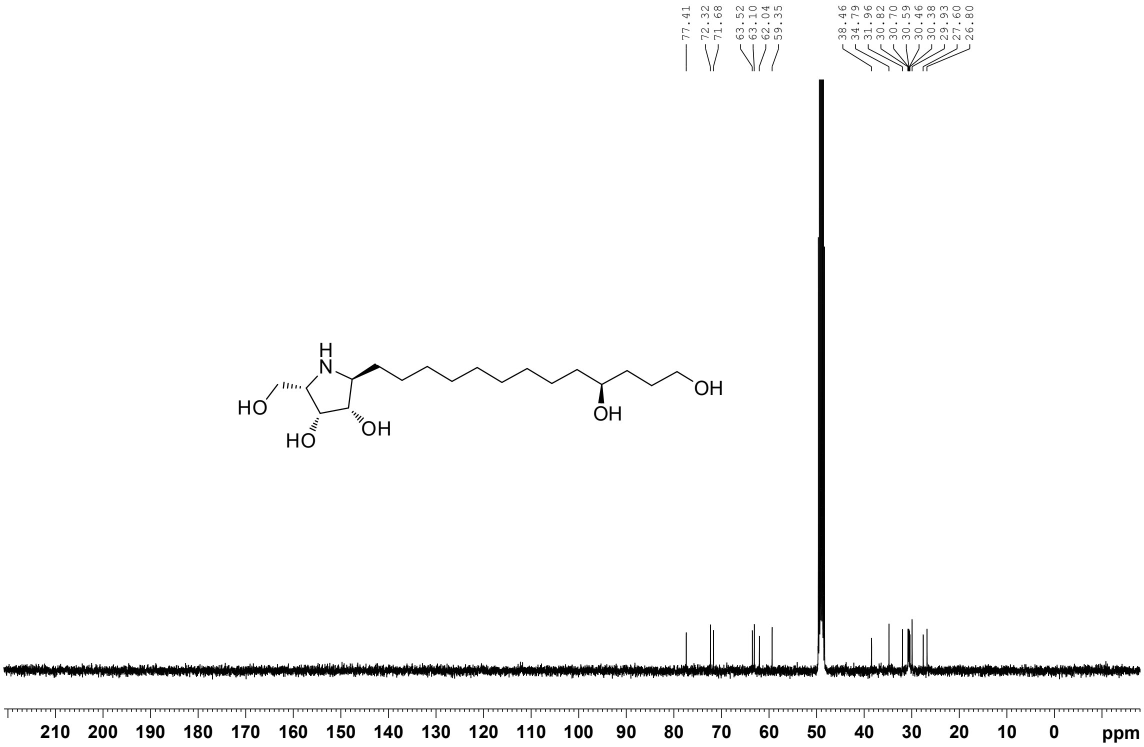

Figure S60

Compound *ent*-3-*epi*-3

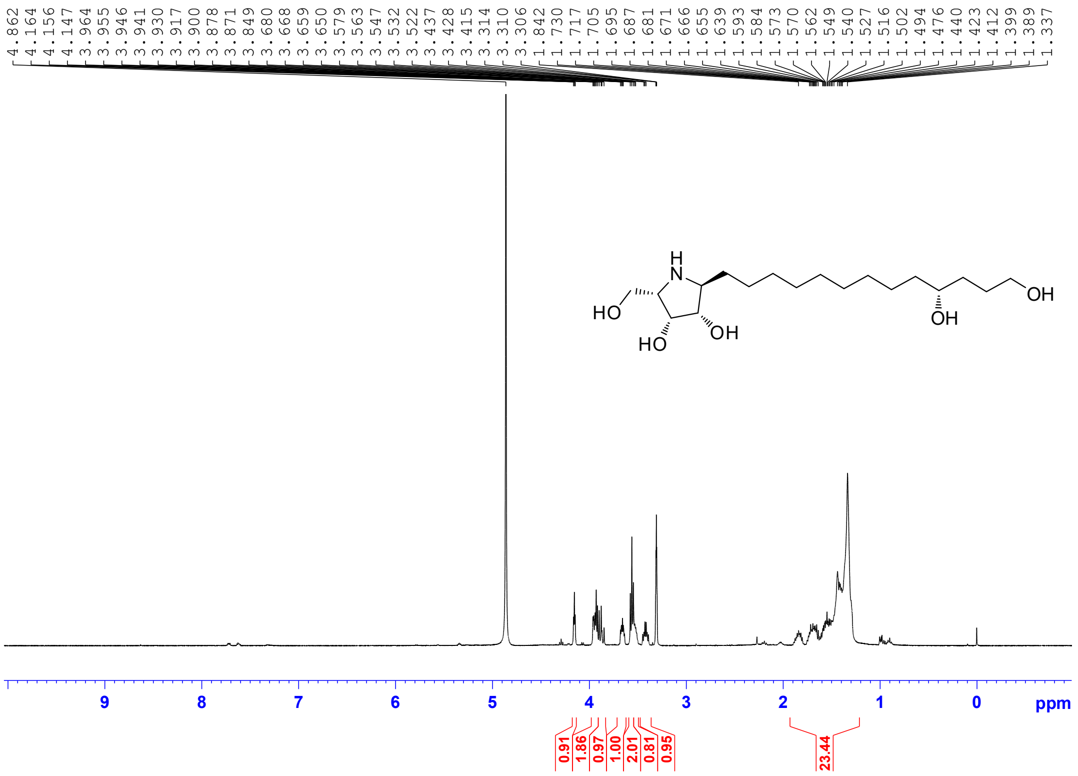

Figure S61

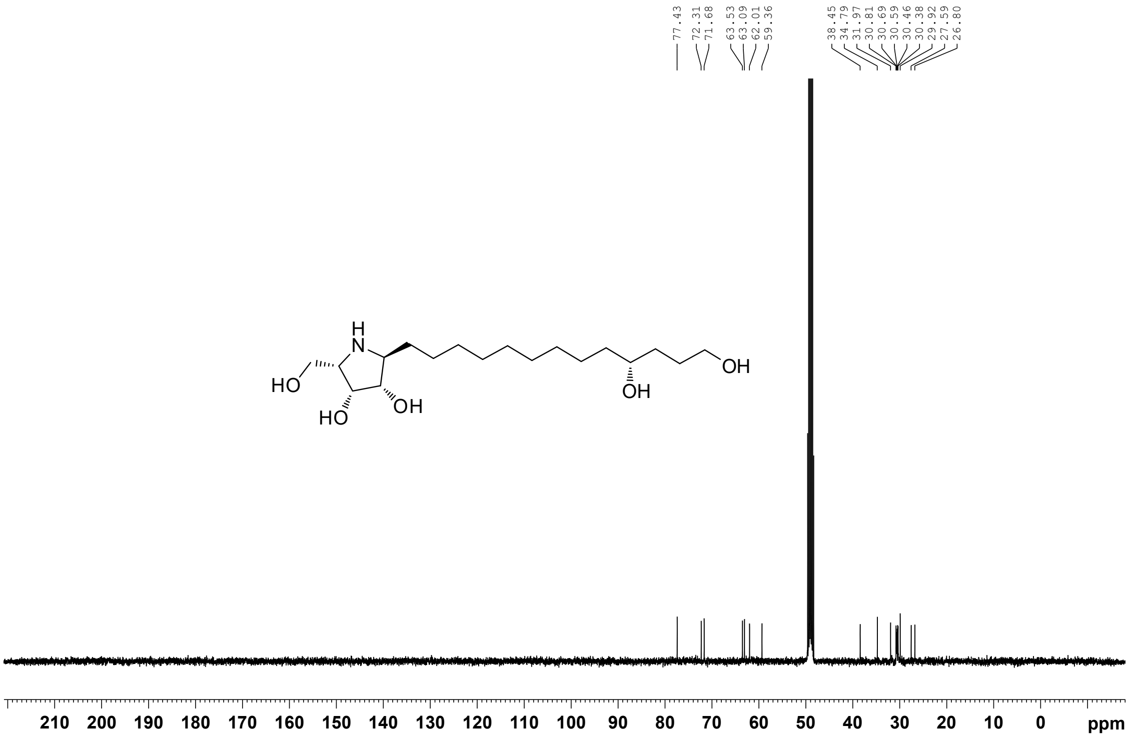

Figure S62

Compound 2-*epi*-3

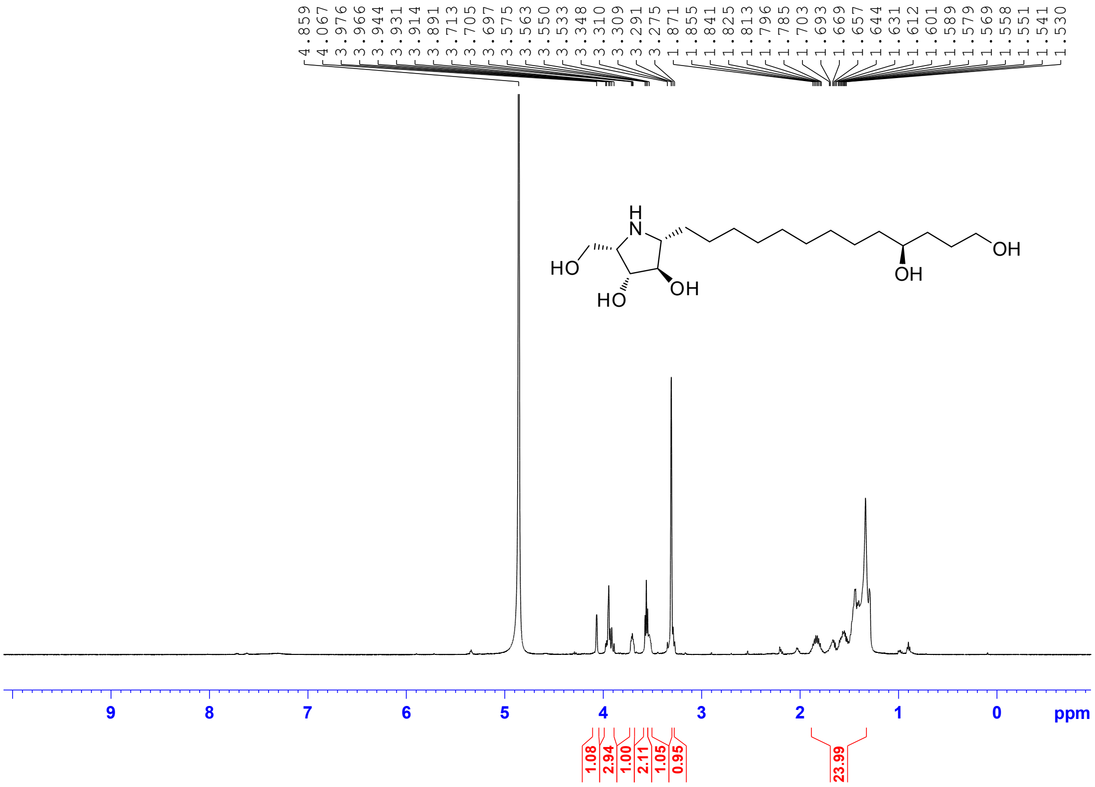

Figure S63

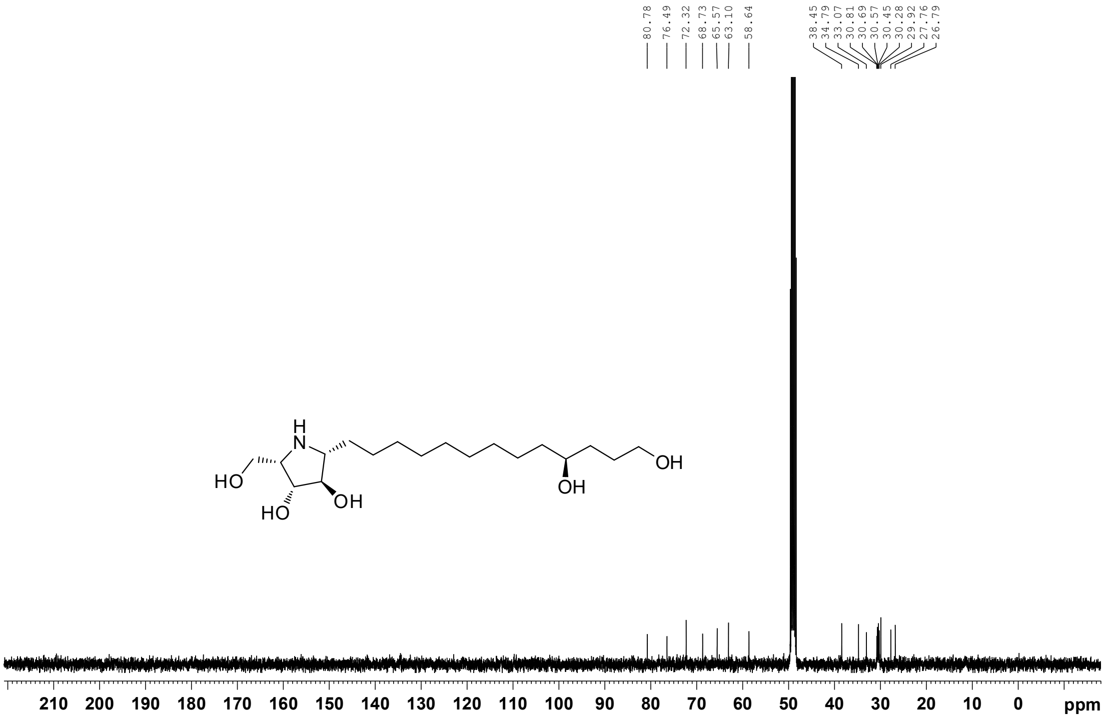

Figure S64

Compound 2,10'-di-*epi*-3

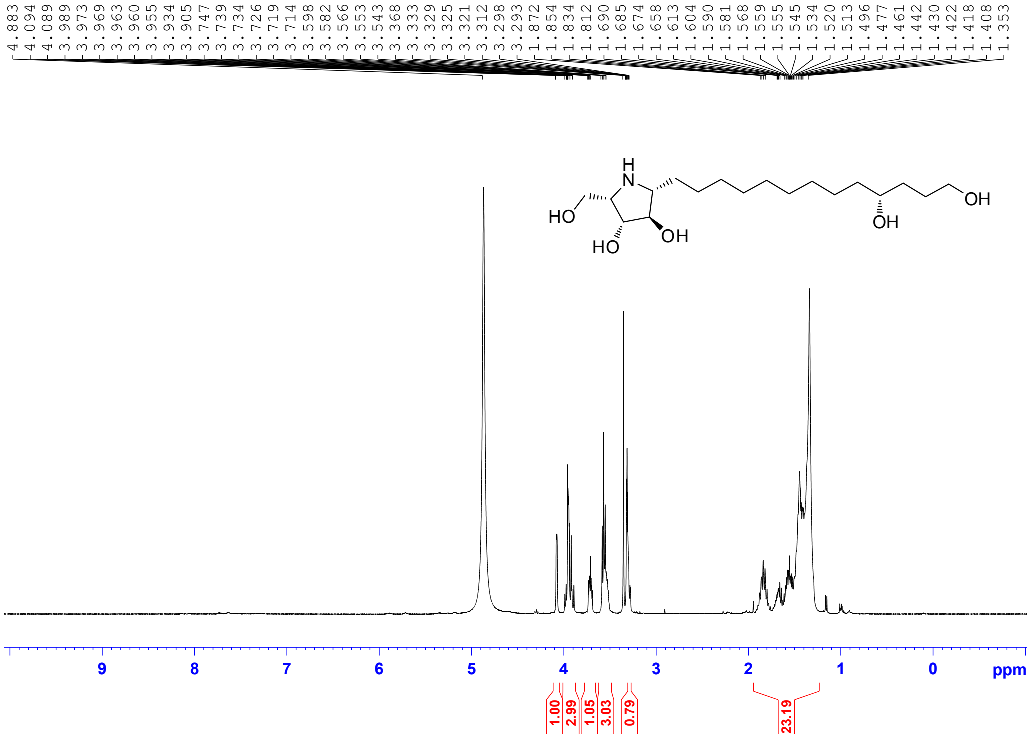

Figure S65

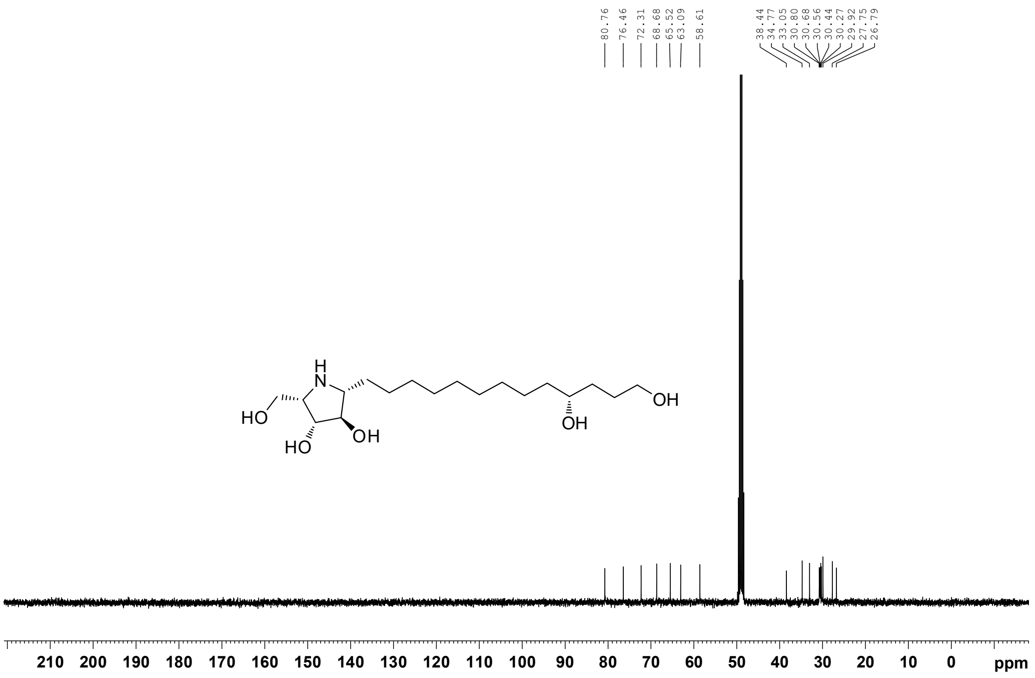

Figure S66

HPLC Analysis of Compounds 15 and *ent*-15

Compound 15

Data File C:\CHEM32\1\DATA\WQK\1509.D
Sample Name: 150901

===

Acq. Operator : WQK
Acq. Instrument : Instrument 1 Location : Vial 1
Injection Date : 2016-9-28 9:04:04
Acq. Method : C:\CHEM32\1\METHODS\JWQ20121205.M
Last changed : 2016-9-28 9:02:24 by WQK
(modified after loading)
Analysis Method : C:\CHEM32\1\METHODS\JWQ20121205.M
Last changed : 2016-9-28 10:26:48 by WQK
(modified after loading)
Sample Info : OD-H H/I=95:5 1 ml/min, 220nm

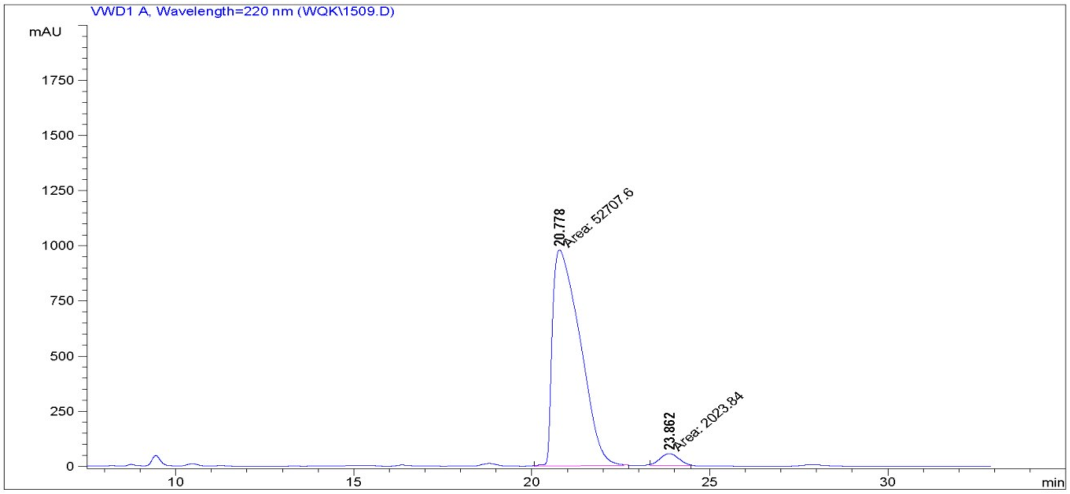

===

Area Percent Report

===

Sorted By : Signal
Multiplier : 1.0000
Dilution : 1.0000
Sample Amount : 1.00000 [ng/ul] (not used in calc.)
Use Multiplier & Dilution Factor with ISTDs

Signal 1: VWD1 A, Wavelength=220 nm

Peak #	RetTime [min]	Type	Width [min]	Area mAU *s	Height [mAU]	Area %
1	20.778	MM	0.8965	5.27076e4	979.92041	96.3022
2	23.862	MM	0.6126	2023.84094	55.06227	3.6978

Totals : 5.47315e4 1034.98268

===

*** End of Report ***

Instrument 1 2016-9-28 10:27:28 WQK Page 1 of 1

Figure S67

Compound *ent*-15

```
Data File C:\CHEM32\1\DATA\WQK\ENT1509.D
 Sample Name: ent150901

    =====================================================================
    Acq. Operator   : WQK
    Acq. Instrument : Instrument 1                    Location : Vial 1
    Injection Date  : 2016-9-28 9:38:39
    Acq. Method     : C:\CHEM32\1\METHODS\JWQ20121205.M
    Last changed    : 2016-9-28 9:02:24  by WQK
                      (modified after loading)
    Analysis Method : C:\CHEM32\1\METHODS\JWQ20121205.M
    Last changed    : 2016-9-28 10:31:20  by WQK
                      (modified after loading)
    Sample Info     : OD-H H/I=95:5  1 ml/min, 220nm
```

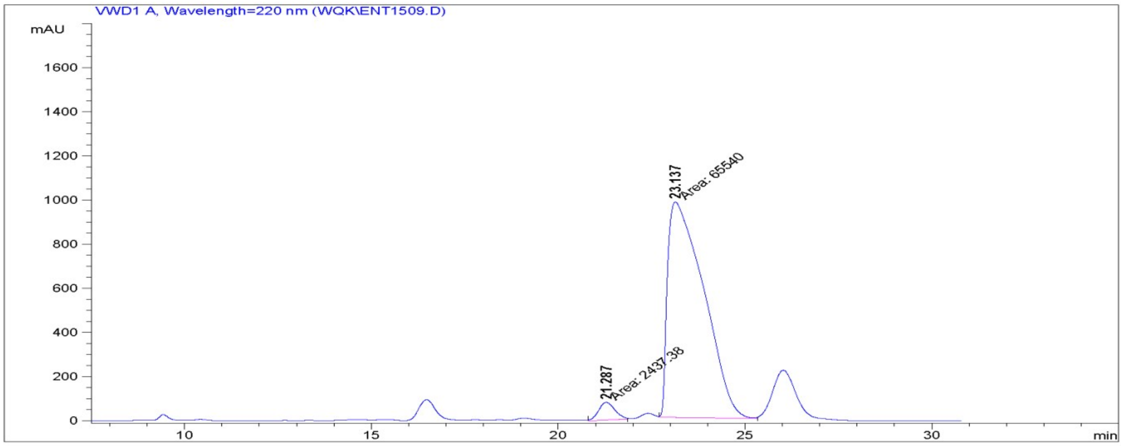


```
    =====================================================================
                         Area Percent Report
    =====================================================================

    Sorted By             :      Signal
    Multiplier            :      1.0000
    Dilution              :      1.0000
    Sample Amount         :      1.00000  [ng/ul]   (not used in calc.)
    Use Multiplier & Dilution Factor with ISTDs

    Signal 1: VWD1 A, Wavelength=220 nm

    Peak RetTime Type  Width     Area      Height     Area
      #   [min]        [min]   mAU   *s    [mAU   ]     %
    ----|-------|----|-------|----------|----------|--------|
       1  21.287 MM    0.5060 2437.37939   80.27544   3.5856
       2  23.137 MM    1.1179 6.55400e4   977.14410  96.4144

    Totals :                  6.79774e4  1057.41954

    =====================================================================
                         *** End of Report ***
```

Instrument 1 2016-9-28 10:31:34 WQK   Page   1 of 1

Figure S68
